# IL-6 controls susceptibility to helminth infection by impeding Th2 responsiveness and altering the Treg phenotype in vivo

**DOI:** 10.1002/eji.201343746

**Published:** 2013-10-08

**Authors:** Katherine A Smith, Rick M Maizels

**Affiliations:** Institute of Immunology and Infection Research, University of EdinburghUnited Kingdom

**Keywords:** IL-6, Parasite infection, Th2 response, Treg cells

## Abstract

IL-6 plays a pivotal role in favoring T-cell commitment toward a Th17 cell rather than Treg-cell phenotype, as established through in vitro model systems. We predicted that in the absence of IL-6, mice infected with the gastrointestinal helminth *Heligmosomoides polygyrus* would show reduced Th17-cell responses, but also enhanced Treg-cell activity and consequently greater susceptibility. Surprisingly, worm expulsion was markedly potentiated in IL-6-deficient mice, with significantly stronger adaptive Th2 responses in both IL-6^−/−^ mice and BALB/c recipients of neutralizing anti-IL-6 monoclonal Ab. Although IL-6-deficient mice showed lower steady-state Th17-cell levels, IL-6-independent Th17-cell responses occurred during in vivo infection. We excluded the Th17 response as a factor in protection, as Ab neutralization did not modify immunity to *H. polygyrus* infection in BALB/c mice. Resistance did correlate with significant changes to the associated Treg-cell phenotype however, as IL-6-deficient mice displayed reduced expression of Foxp3, Helios, and GATA-3, and enhanced production of cytokines within the Treg-cell population. Administration of an anti-IL-2:IL-2 complex boosted Treg-cell proportions in vivo, reduced adaptive Th2 responses to WT levels, and fully restored susceptibility to *H. polygyrus* in IL-6-deficient mice. Thus, in vivo, IL-6 limits the Th2 response, modifies the Treg-cell phenotype, and promotes host susceptibility following helminth infection.

## Introduction

Interleukin-6 (IL-6) is a pleiotropic cytokine produced by multiple cell types and with wide-ranging functions and actions. As well as playing a role in the activation and differentiation of macrophages, lymphocytes, and the terminal differentiation of B cells, IL-6 also actively regulates acute and chronic inflammation [1].

Studies in gene-targeted mice have revealed that IL-6 is important in restraining the acute local and systemic inflammatory response following exposure to endotoxin [2] while reducing susceptibility to bacterial, viral, and fungal infection [3,4]. IL-6 also has a crucial role in promoting the pathogenesis of chronic conditions such as murine inflammatory bowel disease [5], collagen-induced arthritis [6], and in the development of tumors [7].

IL-6 and IL-6-related cytokine responses are transmitted through gp130, activate the JAK-STAT1/3 pathway, and initiate gene transcription in a range of target cells (reviewed in [8]). Naïve T cells activated in the presence of IL-6 and TGF-β differentiate to Th17 cells to drive experimental autoimmune encephalomyelitis (EAE) and collagen-induced arthritis, both of which are alleviated in IL-6^−/−^ mice (reviewed in [9]). Ab-mediated IL-6 blockade has also been shown to inhibit EAE development by limiting the induction of Ag-specific Th17 cells [10].

In the absence of IL-6, naïve T cells activated with IL-2 and TGF-β become Foxp3^+^ peripherally derived Treg (pTreg) cells [11]. The production of IL-6 from activated DCs has been shown to inhibit Treg-cell function [12], and expansion [13] or even induce thymus-derived Treg (tTreg) cells to become Th17 cells in the presence of TGF-β [14]. In some settings, IL-6 can also promote Th2-cell differentiation [15] although its absence does not affect the development of Th2 responses to schistosome eggs [16,17].

In this study, we examined the contribution of IL-6 to the inflammatory and immunoregulatory response generated following infection with the Th2-cell and Treg-cell-inducing gastrointestinal helminth *Heligmosomoides polygyrus* [18,19]. Our results revealed that IL-6 determines susceptibility to helminth infection by modifying the phenotype of the Treg-cell population and limiting protective Th2 responsiveness. Early stimulation of Treg-cell populations in the absence of IL-6 was crucial in regulating excessive pro-inflammatory responses and preventing resistance to helminth infection.

## Results

### IL-6 deficiency confers enhanced resistance to chronic helminth infection

**Figure 1:**
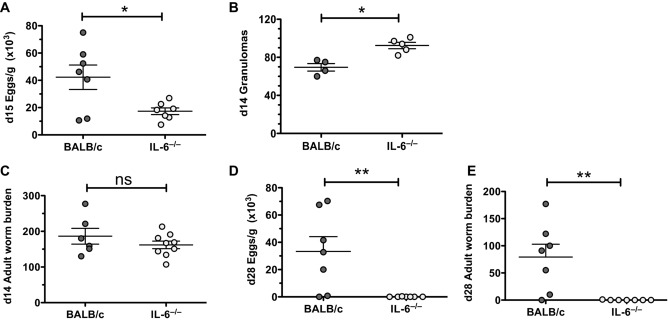
Phenotype of IL-6-deficient BALB/c mice infected with *H. polygyrus*. (A) Egg burdens in BALB/c and IL-6^−/−^ mice 15 days postinfection with *H. polygyrus* (Hp) are shown. (B) Day 14 intestinal granulomas are shown. (C) Day 14 worm burden is shown. (D) Day 28 egg burdens are shown. (E) Day 28 worm burden in *H. polygyrus-*infected BALB/c and IL-6^−/−^ mice are shown. Symbols represent individual mice and data are from one experiment representative of four experiments performed; *p < 0.05, **p < 0.01, unpaired *t* test.

In order to assess the contribution of IL-6 to chronic helminth immunity in a finely balanced Th2/Treg setting, we first determined the survival of adult worms and the production of eggs as a measure of fitness over a 28-day period in IL-6-deficient and IL-6-sufficient BALB/c mice infected with *H. polygyrus*. After the first 14 days of infection, following emergence of the adult worm into the lumen, we found a significant reduction in egg burdens, a significant increase in intestinal granulomas as well as elevated Th2 responses in infected IL-6^−/−^ mice (Fig. 1A and B and data not shown), although adult worm burdens did not differ at this time point (Fig. 1C). At day 28 following infection, when gradual expulsion of the adult worm has begun in BALB/c mice, striking reductions in both egg and adult worm numbers were observed in IL-6^−/−^ hosts, compared with those of their BALB/c counterparts (Fig. 1D and E) and although granuloma numbers had decreased in frequency in both strains, there remained significantly more in the IL-6^−/−^ host, correlating with elevated Th2 responses in these mice (data not shown).

### IL-6-deficient mice display a more potent adaptive Th2 response following helminth infection

**Figure 2:**
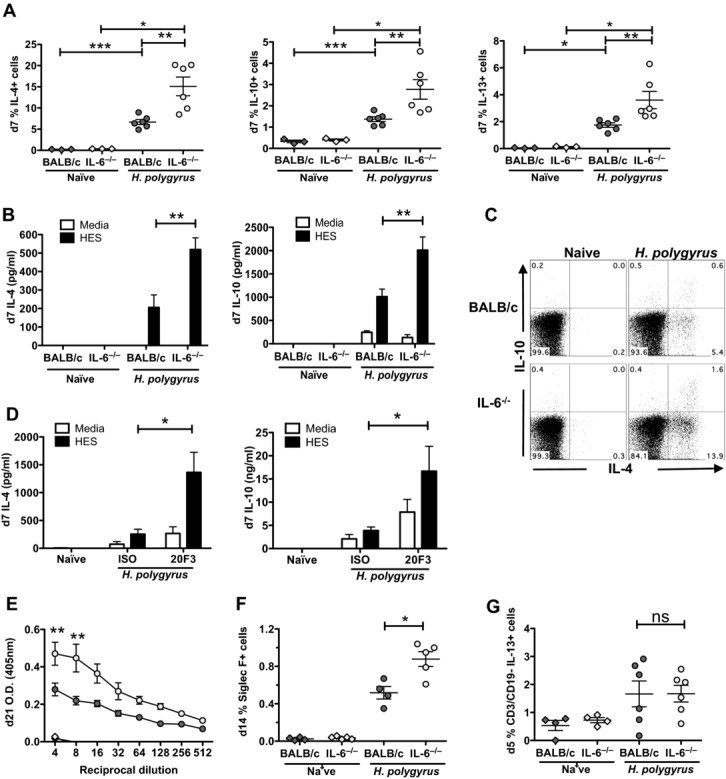
Adaptive Th2 responses to *H. polygyrus* in IL-6-deficient mice, or BALB/c mice treated with anti-IL-6 Ab. (A) IL-4, IL-10, and IL-13 expression by CD4^+^ BALB/c and IL-6^−/−^ MLNCs 7 days postinfection was determined by intracellular staining. (B) IL-4 and IL-10 release from media- or HES-stimulated day 7 BALB/c and IL-6^−/−^ MLNCs was determined by ELISA. (C) Co-expression of IL-4 and IL-10 by day 7 BALB/c and IL-6^−/−^ MLNCs was determined by flow cytometry. (D) IL-4 and IL-10 release from media- or HES-stimulated *H. polygyrus*-infected BALB/c MLNCs treated with 200 μg of a neutralizing anti-IL-6 Ab or a rat IgG control on days 0, 2, 4, and 6 postinfection was determined by ELISA. (E) Day 21 serum Ag-specific IgE to HES in naïve (diamond symbol) and infected (circle symbol) BALB/c (dark gray) and IL-6^−/−^ (light gray) mice is shown. (F) Day 14 MLNC eosinophilia in BALB/c and IL-6^−/−^ mice is shown. (G) Day 5 intracellular staining of MLNC nuocyte populations (CD3^−^CD19^−^IL-13^+^ cells) in BALB/c and IL-6^−/−^ mice are shown. Symbols represent individual mice and data are from one experiment representative of two experiments performed; *p < 0.05, **p < 0.01, ***p < 0.001, one-way ANOVA.

Given the role of IL-4 and IL-13 in mediating helminth expulsion [20] and the contribution of innate lymphoid and adaptive T-cell populations to the production of these cytokines following helminth infection [21], we hypothesized that the late phase of worm expulsion would be determined by the balance of regulatory and effector (Treg:Teff) T-cell responses established in the initial priming phases of infection. The increased number of intestinal granulomas in IL-6^−/−^ mice also indicated potentiation of type 2 responses early in infection, as these are foci of alternatively activated macrophages, which form in an IL-4Rα-dependent manner [22]. To characterize the Treg:Teff dynamic, we performed a number of measures of the innate and adaptive type-2 response. On day 7 following *H. polygyrus* infection, CD4^+^ mesenteric lymph node cells (MLNCs) from IL-6^−/−^ mice expressed higher levels of the Th2 cytokines IL-4, IL-13, and the regulatory cytokine IL-10 by intracellular staining (Fig. 2A) and higher levels of IL-4 and IL-10 following Ag-specific restimulation (Fig. 2B). In WT mice, >50% of IL-10^+^ T cells were also producing IL-4 (Fig. 2C), reflecting the integral part IL-10 plays in both the induction and expression of the Th2 response to helminths [23]. In IL-6^−/−^ mice, an even greater proportion of IL-10 is co-expressed with IL-4, indicating again an intensification of Th2 responsiveness in the absence of IL-6.

To establish that the phenotype of the IL-6^−/−^ mice was directly attributable to the actions of IL-6 and not due to other hematological changes known to occur in the IL-6^−/−^ strain [24], we also depleted WT BALB/c mice with the anti-IL-6 monoclonal Ab 20F3 and found that Ag-specific Th2 responses to infection (as measured by IL-4 and IL-10) were elevated in treated mice MLNCs (Fig. 2D).

IL-6 has been shown to play an important role in driving terminal B-cell differentiation [1], and we therefore assessed the longer term development of Ag-specific Ab production in the sera of BALB/c and IL-6-deficient mice. By day 21 IL-6^−/−^-infected mice developed much higher Ag-specific IgE levels (Fig. 2E), whereas levels of *H. polygyrus* excretory-secretory antigens (HES)-specific IgM, IgG1, and IgG2a were unaffected (data not shown).

To next evaluate the impact of IL-6 deficiency on the innate immune response to *H. polygyrus* infection, we then assessed the generation of eosinophilia, which in other helminth infections can occur independently of the adaptive Th2 compartment in *nu*/*nu* [25], STAT-6^−/−^ [26], and RAG-deficient [27] animals. At day 7 and 14, IL-6-deficient mice exhibited higher levels of MLNC eosinophilia than BALB/c mice, consistent with reports that IL-6^−/−^ mice display enhanced lung eosinophilia during *Schistosoma mansoni* infection [28] (Fig. 2F and Supporting Information Fig. 1G). However, the absence of IL-6 did not alter the early day 5 induction of CD3^−^CD19^−^IL-13^+^ type 2 innate lymphoid cell (ILC-2) populations postinfection in the MLNCs, indicating specific effects on Th2 polarization in the absence of IL-6 (Fig. 2G).

### IL-6-independent generation of Th17 responses in vivo following helminth infection

**Figure 3:**
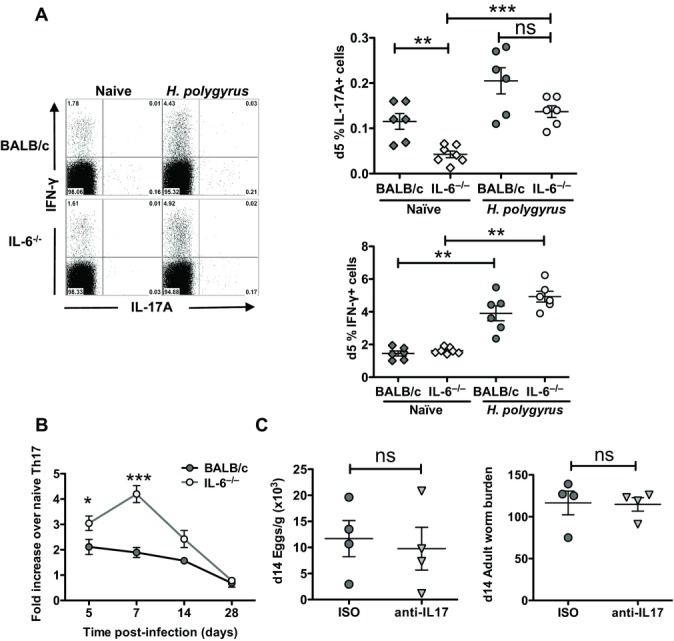
Th17 levels in IL-6-deficient mice following *H. polygyrus* infection. (A) Intracellular staining of MLNCs from naïve and 5-day *H. polygyrus*-infected BALB/c and IL-6^−/−^ CD4^+^ T cells for IL-17A and IFN-γ. (B) The fold increase in Th17 cells by intracellular staining of MLNCs from infected BALB/c and IL-6^−/−^ mice compared with average naïve levels at the respective time-points is shown. (C) Egg and worm burdens at day 14 of *H. polygyrus* infection in BALB/c mice treated with 50 μg of neutralizing anti-IL-17 Ab, or rat IgG2a control, at days 0, 3, 6, and 9 postinfection, are shown. Data shown are (A, B) pooled from two independent experiments each with *n* ≥ 3 mice/group or (C) from one experiment representative of two performed; *p < 0.05, **p < 0.01, ***p < 0.001, one-way ANOVA (A), unpaired *t* test (B, C).

IL-6 is well known as a promoter of Th17 differentiation in settings such as autoimmunity in mice [10]. In the absence of IL-6, the balance between Th17 and Th2 development may be altered, and we therefore compared Th17-cell frequencies in MLNC from BALB/c and IL-6-deficient mice at steady state and days 5, 7, 14, and 28 following *H. polygyrus* infection. Naïve IL-6^−/−^ mice had fewer Th17 (CD4^+^IL-17^+^) cells than BALB/c mice, as observed in mice lacking IL-6 gp130 signaling [29] whereas levels of CD4^+^IFN-γ^+^ cells were similar between genotypes (Fig. 3A). Despite this deficiency, following day 5 *H. polygyrus* infection of IL-6^−/−^ mice, there was a significant outgrowth of Th17 cells to levels similar to those in infected BALB/c mice (Fig. 3A), reflecting a greater fold increase of Th17 cells in IL-6-deficient than in sufficient mice following helminth infection (Fig. 3B). A similar IL-6-independent expansion of MLNC Th17 cells occurred following infection with another gastrointestinal nematode parasite, *Nippostrongylus brasiliensis* (Supporting Information Fig. 1A). Hence, following helminth infection, similar levels of Th17 cells are seen in susceptible and resistant genotype hosts.

To identify other potential stimulators of Th17 in the IL-6-deficient setting, we also examined IL-21 [30], IL-23 [31], and IL-1β [32] expression within whole MLNC, each implicated in the development and stabilization of CD4^+^IL-17^+^ T cells in vitro and in mucosal tissues. However, at day 7 following *H. polygyrus* infection, we found neither compensatory upregulation of IL-21 or IL-1β in IL-6-deficient mice, nor did we find dysregulated expression of the IL-23R, responsive to IL-23, by quantitative PCR (Supporting Information Fig. 1B).

The IL-6-independent generation of Th17-cell responses to helminth infections (Fig. 3B) raised the question of the functional contribution of these cells to helminth immunity. We therefore administered anti-IL-17 neutralizing Ab to BALB/c mice and assessed whether this was able to modify egg and worm burdens in vivo. We found that Ab treatment over 14 days did not alter egg or worm burden in BALB/c mice (Fig. 3C), or the Th2 and granulomatous response in vivo (data not shown). Hence, the heightened resistance of IL-6^−/–^ mice cannot be attributed to a pivotal role for IL-17 during infection.

### Altered Foxp3^+^ Treg-cell phenotype in *H. polygyrus-*infected IL-6-deficient and IL-6-depleted mice

A further subset of T cells, which is prominent during *H. polygyrus* infection is the Foxp3^+^ Treg-cell population [18], whose suppressive function may play an important role in determining the outcome of infection [19]. As depletion of Treg cells during acute *H. polygyrus* infection of Foxp3-diphtheria toxin receptor-expressing DEREG mice resulted in an amplified antiparasite Th2 response [33], we considered the possibility that aberrant Treg-cell development permitted a stronger and protective Th2 arm to evolve in IL-6-deficient mice. Altered Treg-cell expression could also arise in mice lacking IL-6 given the dominant role this cytokine is reported to play in inhibiting Foxp3^+^ T-cell induction following immunization in vivo or in the presence of TGF-β in vitro [9].

**Figure 4:**
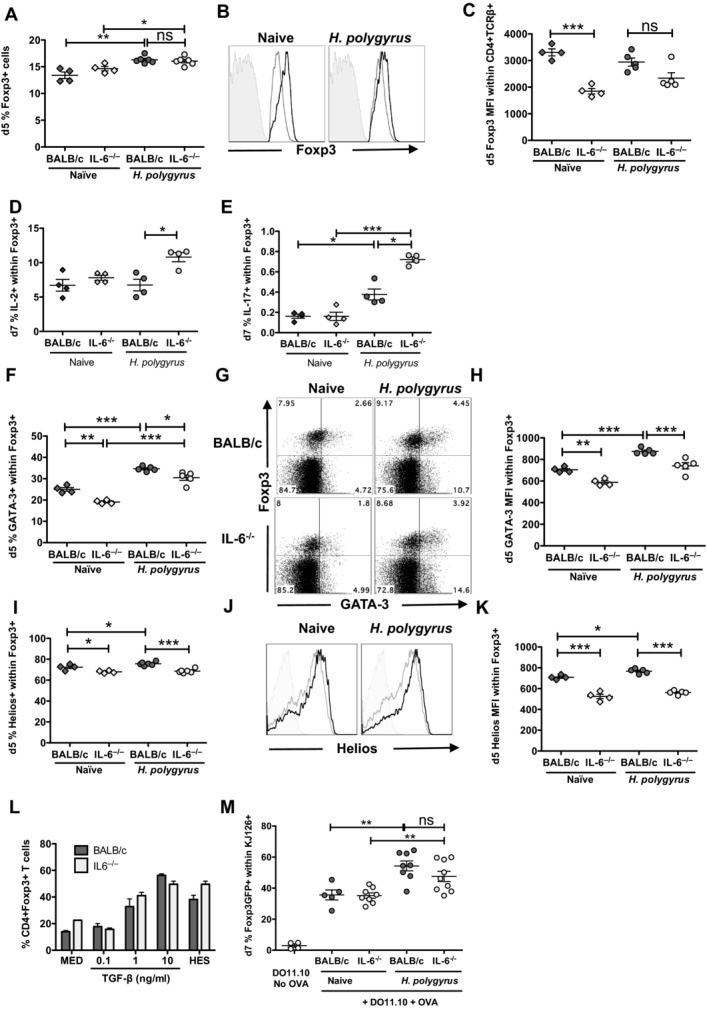
Treg-cell phenotype in vivo in IL-6-deficient mice following *H. polygyrus* infection. (A) The frequency of Foxp3^+^ cells in MLNC CD4^+^ populations of naïve BALB/c or IL-6^−/−^ mice and at day 5 postinfection with *H. polygyrus* is shown. (B) Representative histogram of MLNC CD4^+^ Foxp3 expression in *H. polygyrus*-infected BALB/c (black line histogram) and IL-6^−/−^ (gray line histogram) mice. Isotype control represented by filled gray histogram. (C) Mean fluorescence intensity (MFI) of Foxp3 expression in naïve and day 5-infected CD4^+^TCRβ^+^ MLNCs is shown. (D) The frequency of IL-2^+^ cells within CD4^+^Foxp3^+^ MLNCs of naïve or 7-day infected BALB/c and IL-6^−/−^ mice is shown. (E) The frequency of IL-17^+^ cells within CD4^+^Foxp3^+^ MLNCs of naïve or 7-day infected BALB/c and IL-6^−/−^ mice is shown. (F) The frequency of GATA-3 expression in CD4^+^Foxp3^+^ MLNCs of naïve or 5-day infected BALB/c and IL-6^−/−^ mice is shown. (G) Representative flow cytometry plot of MLNC CD4^+^ Foxp3 and GATA-3 expression in naïve and 5-day *H. polygyrus*-infected BALB/c and IL-6^−/−^ mice is shown. (H) MFI of GATA-3 expression in the same cell populations as (G). (I) The frequency of Helios^+^ cells within the same cell populations as (G) is shown. (J) Representative histogram of Helios expression within CD4^+^Foxp3^+^ MLNCs, colored as in (B) is shown. (K) MFI of Helios expression is shown. (A–K) Data shown are from one experiment representative of three performed with ≥4 mice per group. (L) Proportion of Foxp3^+^ cells among naïve BALB/c and IL-6^−/−^ CD4^+^ cells stimulated in vitro with 1 μg plate-bound anti-CD3/CD28 and 20 ng/mL rIL-2 for 72 h at 37°C/5% CO_2_ is shown. One experiment representing three in vitro repeats with duplicate or triplicate wells for each condition is shown. (M) Proportion of CD4^+^KJ126^+^Foxp3GFP^+^ MLNCs derived in vivo following CD4^+^Foxp3GFP^−^ FoxDO11.10 transfer and administration of 1% OVA protein in the water at day 7 postinfection or in naïve mice is shown. Each symbol represents an individual mouse and data were pooled from two in vivo experiments; *p < 0.05, **p < 0.01, ***p < 0.001, one-way ANOVA.

When the MLNC CD4^+^ T-cell compartment was analyzed for expression of the Treg-cell marker Foxp3, similar proportions were found in naïve BALB/c and IL-6-deficient mice, as also noted in mice impaired in gp130 signaling [29] (Fig. 4A); in both genotypes, *H. polygyrus* infection stimulates a small but significant increment in the percentage of Foxp3^+^ T cells.

The expression of Foxp3 is strongly associated with Treg-cell activity and repression of effector CD4^+^ T-cell lineage differentiation [34]. Notably, the expression intensity of Foxp3 was significantly lower in naïve IL-6^−/−^ compared with that in BALB/c mice, while the disparity between the strains narrowed following infection (Fig. 4B and C).

The plasticity of Foxp3^+^ cells is being increasingly recognized, and the deletion of Foxp3 expression can result in loss of suppressive function and acquisition of pro-inflammatory cytokine production, particularly IL-2 and IFN-γ [35] while in vitro, IL-6 can reprogram fully differentiated Treg cells toward the Th17 lineage [14,36]. In order to assess the impact of IL-6 deficiency on Treg-cell function in vivo following helminth infection, we performed Foxp3 staining in concert with intracellular cytokine staining at a time-point when effector cell responses were dysregulated in IL-6^−/−^ mice (day 7). While IL-2 and IL-17 expression by MLNC Foxp3^+^ Treg cells was very low in both genotypes of naive mice, infection induced a significant increase in Foxp3^+^IL-17^+^ cell numbers in the BALB/c strain. Infected IL-6^−/−^ mice, moreover, showed raised IL-2 expression among the Foxp3^+^ population, which also displayed significantly higher IL-17 production compared to BALB/c mice (Fig. 4D and E).

We next tested expression of the transcription factor GATA-3 at steady state and in infected mice, which has recently been recognized to control both Foxp3 expression in vivo [37] and Foxp3^+^ T-cell fate and function [38]. In the absence of IL-6, significantly fewer MLNC Treg cells expressed GATA-3 (Fig. 4F and G), and in particular expression levels (as measured by intensity of GATA-3 staining within Foxp3^+^ T cells) were significantly diminished both in steady state and in response to infection at a time-point preceding effector cell dysregulation (day 5; Fig. 4H). Although the proportion of GATA-3^+^ Treg cells increases with infection in both strains, the proportion of GATA-3^+^ Treg cells and expression of GATA-3 within the MLNC Treg-cell population remains significantly lower in IL-6^−/−^ mice.

Another transcription factor, Helios, has been closely associated with Treg cells, having first been used to distinguish thymic Treg cells from Foxp3^+^Helios^−^ peripherally derived Treg cells (pTreg cells) [39]. However, Helios may also be expressed during activation of Foxp3^−^ T cells [40] while very recent studies point to a role in stabilizing Foxp3 expression in human Treg cells [41]. Hence, Helios^−^ cells have greater potential to replace Foxp3 expression with that of effector cytokines such as IL-2, IL-17, and IFN-γ [39]. Moreover, transgenic overexpression of IL-6 in vivo significantly reduces the frequency of Foxp3^+^Helios^−^ cells, suggesting a link with Helios expression [42]. In accordance with these data, we found significantly lower levels of Helios expression within the MLNC Foxp3^+^ Treg-cell population of IL-6-deficient mice, both in terms of proportion (Fig. 4I) and staining intensity (Fig. 4J and K), which were not recovered 5 days after *H. polygyrus* infection. Interestingly, we also found that Helios expression by MLNC Foxp3^+^ cells correlated with GATA-3, CD45RB, OX-40, and Foxp3 expression as well as Ki67 (a marker of proliferation) but not CD44, CD25, or ICOS. Hence, Helios may more reliably mark Foxp3^+^ Treg-cell stability and function in vivo (Supporting Information Fig. 1C and D) rather than activation status per se.

In order to address whether induced Treg cells were aberrent in IL-6^−/−^ mice following *H. polygyrus* infection, independently of Helios expression, we assessed the ability to induce Foxp3 expression in purified CD4^+^ T cells cultured in the presence of anti-CD3/CD28, IL-2, and TGF-β; we also tested HES, which we previously demonstrated can mimic TGF-β as a Treg-cell-driving agent [19]. As shown in Fig. 4L, cells from both strains were able to respond similarly and increase the proportion of CD4^+^Foxp3^+^ T cells under Treg-cell-inducing conditions. To confirm that Treg-cell induction was similar within an in vivo setting, we also tested de novo Ag-specific Treg-cell induction in vivo by transferring FACS-sorted Fox.DO11.10 CD4^+^GFP^−^ cells into BALB/c and IL-6^−/−^ mice in the absence and presence of *H. polygyrus* infection and administered soluble OVA protein orally [19]. Following infection, an increase in the proportion of MLNC Foxp3^+^CD4^+^ cells within the transferred Fox.DO11.10 population occurred in both strains to a similar extent (Fig. 4M). These results indicate that loss of Treg-cell function may specifically occur within the tTreg-cell population in mice deficient in IL-6.

### Rescue of Foxp3^+^ Treg-cell phenotype and reversion to susceptibility by IL-2:anti-IL-2 treatment

**Figure 5:**
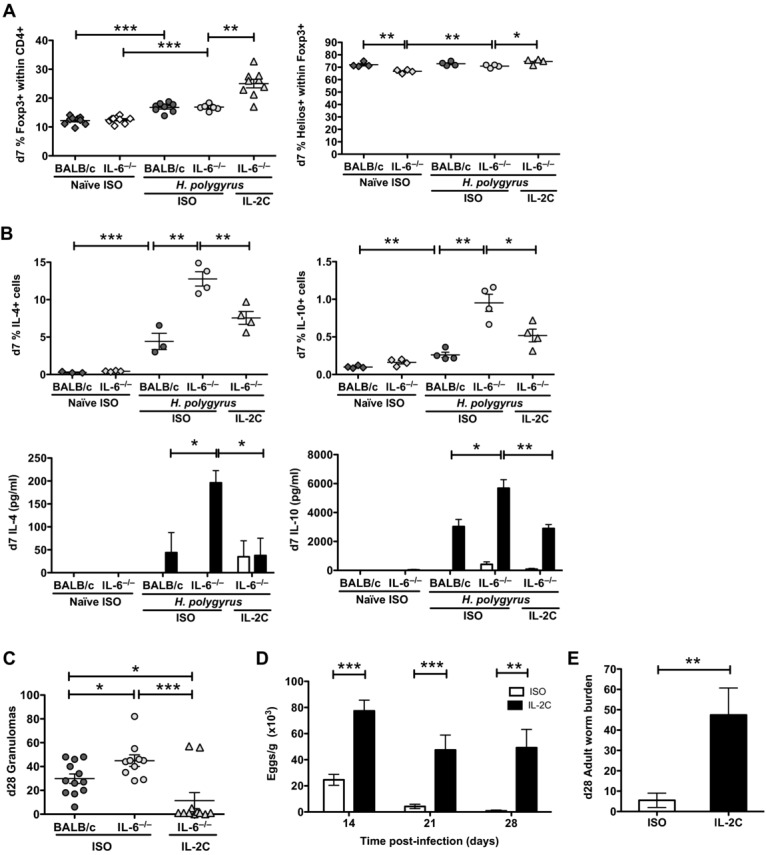
Treg-cell phenotype and helminth survival in IL-6-deficient mice treated with anti-IL-2:IL-2 complex (A) Percentages of Foxp3^+^ within CD4^+^ MLNCs (left) and Helios^+^ T cells within Foxp3^+^ (right) in naïve and at day 7 postinfection *H. polygyrus*-infected BALB/c and IL-6^−/−^ mice given 25 μg isotype control (ISO) or a complex of rmIL-2 (2.5μg):α-IL-2m Ab (25 μg; IL-2C) immediately after infection are shown. (B) The frequency of IL-4 and IL-10 expression following intracellular staining of CD4^+^ MLNCs (top) or IL-4 and IL-10 release from media or HES-stimulated whole MLNC cultures (bottom) at day 7 postinfection in naïve and *H. polygyrus*-infected BALB/c and IL-6^−/−^ mice treated with an isotype control (ISO) or IL-2:anti-IL-2 complex (IL-2C) immediately after infection are shown. Symbols represent individual mice and data shown are from one experiment representative of two replicate experiments performed. (C) The number of day 28 intestinal granulomas in *H. polygyrus*-infected BALB/c and IL-6^−/−^ treated with ISO or IL-2C are shown. (D) Egg burdens over time in IL-6^−/−^ mice treated with ISO or IL-2C are shown. (E) Adult worm burdens at day 28 postinfection in IL-6^−/−^ mice treated with ISO or IL-2C are shown. (C–E) Symbols represent individual mice and data shown were pooled from three experiments with *n* > 5 mice/group; *p < 0.05, **p < 0.01, ***p < 0.001, one-way ANOVA (A–C), unpaired *t* test (D, E).

To test the hypothesis that mice lacking IL-6 have an impaired CD4^+^Foxp3^+^ Treg-cell compartment, we tested the effect of selectively boosting this population through administration of an IL-2:anti-IL-2 complex, which has been found by other investigators to expand CD4^+^Foxp3^+^ Treg cells in vivo [43–45], stabilize Treg-cell Foxp3 expression [46,47], and increase GATA-3 expression [38]. One intraperitoneal injection of the complex immediately following infection with *H. polygyrus* resulted in a dramatic increase in the percentage of Foxp3^+^ Treg cells and increased expression of Helios on Foxp3^+^ T cells in the MLNCs of IL-6-deficient mice by day 7 postinfection (Fig. 5A).

In accordance with reports that the IL-2:anti-IL-2 complex stabilizes Foxp3 expression [47], and that CD25^+^ Treg cells are less prone to switch into effector mode [48] we find that administration of the complex significantly reduces MLNC cytokine production by Foxp3^+^ T cells in terms of IL-10, with a downward trend for IL-2 production, without affecting IL-17 (Supporting Information Fig. 1E).

Treg-cell expansion and stabilization was associated with suppression of higher Th2 responses in the IL-6-deficient mice, as measured both by intracellular cytokine staining of CD4^+^ MLNCs for IL-4, IL-10, and IL-13, as well as Ag-specific restimulation of whole MLNCs (Fig. 5B and Supporting Information Fig. 1F). These changes were reflected in an associated reduction in the granulomatous response in IL-6^−/−^ mice treated with the IL-2C (Fig. 5C) and are consistent with observations in a mouse model of airway allergic inflammation [44]. Interestingly, while IL-2:anti-IL-2 complex in the airway model was reported to suppress lung eosinophilia, helminth-induced eosinophilia in the MLNCs was unaffected by administration of the complex (Supporting Information Fig. 1G).

Most importantly, administration of IL-2:anti-IL-2 complex also evoked a dramatic switch in infection status. From 14 days postinfection, treated mice showed greatly increased egg burdens through to 28 days postinfection (Fig. 5D) at which time substantially higher worm burdens had persisted (Fig. 5E). Hence the remarkable phenotype of helminth-infected IL-6-deficient mice can be fully reversed by intervention to reinvigorate the Foxp3^+^ Treg-cell compartment.

## Discussion

IL-6 plays many crucial roles in the immune system, not least in the differentiation and maturation of different T-cell subsets [11,49,50]. Our data show that in the absence of IL-6, more potent Ag-specific Th2 responses can develop resulting in increased immunity and parasite resistance. Immunity was not due to lower proportions of Th17 cells in the MLN in mice lacking IL-6, as these mice were able to generate significantly increased proportions of Th17 cells equivalent to the level of WT mice following infection. Furthermore, administration of a neutralizing anti-IL-17 Ab had no impact on egg burden or worm burden in WT mice. IL-6-deficient mice also had an altered Treg phenotype, expressing lower levels of Foxp3, Helios, and GATA-3 at steady state and producing higher levels of IL-2 and IL-17 following *H. polygyrus* infection. The resistant phenotype of the IL-6-deficient mouse could be fully reversed by administration of an anti-IL-2:IL-2 complex, rescuing the Treg-cell phenotype, inhibiting Ag-specific Th2 responses, and restoring susceptibility to chronic helminth infection.

Previous work had established that mice deficient in IL-6 are not impaired in their ability to produce an in vivo Th2 response following injection of *S. mansoni* eggs [17]. Moreover, Th2 responses are enhanced following mycobacterial vaccination of mice deficient in, or neutralized for, IL-6 [51]. We now demonstrate that enhanced Ag-specific Th2 responses of IL-6-deficient mice can be reproduced in WT mice by administration of neutralizing anti-IL-6 following *H. polygyrus* infection. Interestingly, CD4^+^ T cells within the MLNC are the predominant source of the IL-10 in response to *H. polygyrus* infection, similar to the situation seen in human filariasis infections [52]; however, it remains to be seen whether those CD4^+^IL-10^+^IL-4^+^ co-expressing cells elicited following *H. polygyrus* infection contribute to a similar state of Ag-specific hyporesponsiveness as that apparent in human disease.

Primary and secondary infection with *H. polygyrus* promotes a T-cell-dependent IgE response, which requires IL-4 signaling in vivo [53]. A seminal study demonstrated that IgE deficiency had no impact on protective immunity following secondary challenge with *H. polygyrus*, and that only passive transfer of polyclonal IgG Ab was able to significantly reduce adult worm burden following primary infection [54]. Here, we show that Ag-specific IgE is increased following *H. polygyrus* infection of IL-6-deficient mice, commensurate with increased Th2 responses in the same mice, however given the aforementioned findings, it is unlikely that increased IgE contributes to the improved resistance of these mice following primary infection with *H. polygyrus*. Mice deficient in IL-6 also exhibited increased eosinophilia in the MLN, consistent with reports that IL-6-deficient mice display enhanced lung eosinophilia and parasite mortality following *S. mansoni* infection [28]. In this model, IL-6 was expressed in the pulmonary microvasculature of infected mice, highlighting the importance of cytokine production by the endothelium in mediating parasite clearance.

IL-6 exerts an important influence on Th17-cell differentiation and mediates the dichotomy underlying the generation of pathogenic Th17 and Treg cells induced by TGF-β [11]. As in the case of mice lacking IL-6 gp130 signaling [29], we found that mice lacking IL-6 had lower proportions of Th17 cells in the MLN at steady state. However, following infection with two different parasitic helminths, these mice were able to generate significantly increased percentages of Th17 cells equivalent to the level of infected WT mice. The generation of Th17 cells in an IL-6-independent manner has been described through an IL-21-linked pathway [30] and through microbiota-induced IL-1β in the intestine [32]. Although we found no compensatory upregulation of either of these factors in IL-6-deficient mice by quantitative PCR, it is likely that infection stimulates other factors that may, for example, activate STAT3 through the relatively promiscuous IL-6/IL-12 family of ligands. This may even extend to mediators such as IL-9 [55], which is not only upregulated in helminth infection, but more intensely so in IL-6-deficient mice. These possibilities are currently under investigation in our laboratory.

IL-6 is known to strongly influence the size and nature of the Treg-cell compartment in mice, but in a manner highly dependent upon the context of the inflammatory condition. In graft-versus-host disease, the blockade of IL-6R-mediated signaling increases Treg-cell numbers at the expense of Th1/17, dampening immune reactivity [56]. A similar switch is accompanied by the rapid generation of a strong Th2 response and enhanced immunity to the intestinal nematode *Trichuris muris*, in highly susceptible IL-10-deficient mice in which T cells cannot respond to IL-6 [57]. More recent studies have implicated differential cytokine signal requirements for the generation of pTreg cells and tTreg cells [58] and have suggested that IL-6 may play a role in controlling pTreg-cell generation in vivo at steady state [38,42].

Although Helios has been used as a marker of natural Treg cells, new studies have suggested that its expression more closely reflects the activation status of Treg cells [40]. In this light, we measured CD25, CD44, and ICOS as markers of activation, which might correlate with Helios expression, but found this not to be the case either at steady state or following infection (Supporting Information Fig. 1C and D). Interestingly, Ki67 staining of the Foxp3^+^ Treg-cell population did significantly positively correlate with Helios expression, implying that Helios^+^ cells have a higher constitutive turnover rate in steady state, and are the major regulatory population responding to infection. As IL-6^−/−^ Treg cells expressed lower levels of Helios, our results imply that their proliferation may be impaired, perhaps explaining the more vigorous Th2 responses in these mice.

Expression of Helios also strongly positively correlates with that of CD45RB, GATA-3, and OX40 as well as of Foxp3 itself. Given the critical role Foxp3 plays in the suppressive function of Treg cells in vitro and in vivo [34,59], these results indicate that IL-6 may be required to stabilize Treg-cell function in vivo. This conclusion is supported by lower GATA-3 expression in IL-6-deficient mice, and recent studies highlighting that this transcription factor controls Foxp3 expression and thereby Treg-cell function [37,38]. OX40 also plays an important role in maintaining Treg-cell fitness [60] and it may be that selective loss of OX40 expression on the Treg-cell population in IL-6-deficient mice may render these cells less able to proliferate in response to ligation [61]. Although CD45RB expression also correlated with Helios within the Treg-cell population, as noted previously [40], lower CD45RB expression was also apparent within the Foxp3^−^ population of mice deficient in IL-6, suggesting a global impact on the CD4^+^-cell population, rather than a specific effect on Treg-cell phenotype.

The possibility that a deficiency in IL-6 may destabilize tTreg-cell function in vivo was further tested by de novo induction of Treg cells in vivo and in vitro; as these processes occurred normally in mice deficient in IL-6, destabilization of Treg-cell function must occur within the tTreg-cell population, as postulated elsewhere [42]. The use of an anti-IL-2:IL-2 complex, which can stabilize Foxp3 and GATA-3 expression in vivo [38,42], was able to fully reverse the phenotype of IL-6-deficient mice providing further evidence that defective tTreg-cell function enhances immunity and worm expulsion in these mice. Finally, identifying the major contributor of IL-6 from a diverse range of cell types to this striking phenotype remains a key area of interest for further research in this infection setting.

## Materials and methods

### Mice

BALB/c mice were bred in-house at the University of Edinburgh; IL-6-deficient strains originated from Kopf et al. [3] and were backcrossed to BALB/c by Paul Garside (University of Strathclyde) before being rederived in-house.

### Ethics statement

All animal protocols adhered to the guidelines of the UK Home Office, complied with the Animals (Scientific Procedures) Act 1986, were approved by the University of Edinburgh Ethical Review Committee, and were performed under the authority of the UK Home Office Project Licence number 60/4105.

### Parasites and Ags

*H. polygyrus bakeri* and *N. brasiliensis* were maintained, and adult *H. polygyrus* HES was prepared as previously described [19,23]. Egg burdens of individual mice were assessed by weighing feces before dissolving in 2 mL PBS; following addition of 2 mL saturated sodium chloride solution, egg counts were performed using a McMaster chamber and the average number of eggs/g feces calculated per sample.

### In vivo Ab depletion

A neutralizing anti-IL-6 Ab (Clone MP5–20F3) or rat IgG (purified from sera) were generated in-house and 200 μg injected i.p. on days 0, 2, 4, and 6 postinfection, with cells harvested on day 7. A neutralizing anti-IL-17 Ab (Clone 50104, Cat No MAB421) or an IgG2a control (Clone 54447, Cat No MAB006) were purchased from R&D Systems and 50 μg was injected i.p. on days 0, 3, 6, and 9 postinfection (total 200 μg) [62–64], with cells harvested on day 14.

### Preparation and administration of IL-2/anti-IL-2 complexes

Recombinant murine IL-2 and anti-mouse IL-2 (clone JES6–1A12) were purchased from eBioscience with isotype control (rat IgG2a). Immediately following infection with *H. polygyrus*, mice were injected i.p. with 200 μL PBS solution containing 2.5 μg IL-2 and 25 μg anti-IL-2, which had been prepared and incubated for 30 min at room temperature before delivery.

### In vitro Ag-specific restimulation

A single cell suspension was made of MLN before plating cells at 1 × 10^6^/well in the presence of 2 μg/mL HES and media alone for 72 h at 37°C/5% CO_2_. Supernatants were then harvested and analyzed for IL-4, IL-10, and IL-13 by commercially available ELISA (BD Pharmingen).

### Treg induction

In vivo and in vitro Treg-cell induction was performed as previously described, by adoptive transfer of Foxp3-GFPxDO11.10 T cells into BALB/c mice, and by in vitro stimulation of purified CD4+ T cells under Treg-inducing conditions [19].

### Flow cytometry

All flow cytometry was performed using Becton-Dickinson Canto or LSR-II flow cytometers. For Treg-cell phenotyping, 10^6^ MLN cells were stained with a combination of FITC-conjugated Abs to CD4 or CD25; A700 or PerCP-conjugated CD4 and Biotin anti-CD103 followed by Streptavidin PerCP for 20 min at 4°C. Following fixation and permeabilization using the Foxp3 staining kit (eBioscience), cells underwent intracellular staining with a combination of PE-conjugated Abs to Helios [39] and APC or Pacific Blue-conjugated Abs to Foxp3.

For intracellular cytokine staining, MLNCs were first incubated with 0.5 μg/mL PMA and 1 μg/mL ionomycin for 1 h before the addition of 10 μg/mL Brefeldin A for a further 3 h. Staining was performed by resuspending cells in a combination of FITC conjugated Abs to CD8 or CD3 and CD19; PerCP conjugated anti-CD4 for 20 min at 4°C, washed again then fixed for 20 min with 200 μL Fix/Perm buffer (BD Pharmingen). Fixation buffer was removed with two washes with permeabilization buffer (BD Pharmingen) and samples were split and subsequently stained for intracellular cytokines using 1/200 anti-IFN-γ-allophycocyanin, anti-IL-4-PE, anti-IL-10-allophycocyanin, anti-IL-13-allophycocyanin, anti-IL-17-PE, or the relevant isotype control for 20 min in Perm buffer. Combined cytokine and Foxp3 staining was performed by fixation of cells following surface staining with the Foxp3 staining kit (eBioscience), with all subsequent steps carried out in Foxp3 permeabilization buffer.

### Statistical analysis

Data were assessed for normality and equal variances and were log transformed if required; all data passed these criteria and an unpaired *t* test was used or, where more than three groups were being tested, a parametric one-way ANOVA followed by Tukey's multiple comparison test was used.

## Abbreviations

HES: *Heligmosomoides polygyrus* excretory-secretory antigens
MLNC: mesenteric lymph node cell

## Supporting Information

As a service to our authors and readers, this journal provides supporting information supplied by the authors. Such materials are peer reviewed and may be re-organized for online delivery, but are not copy-edited or typeset. Technical support issues arising from supporting information (other than missing files) should be addressed to the authors.

**Figure S1.** A. Th17 responses during active N. brasiliensis infection in IL-6-deficient BALB/c mice. BALB/c and IL-6 –/– mice were infected with 250 L3 of N. brasiliensis s.c. and 7 days later, MLNC recovered for intracellular cytokine staining and flow cytometry. Representative FACS staining of IL-17 versus IFN-γ (left panel) and percentage of IL-17 + cells (right panel) within the CD4 + population of naïve and N. brasiliensis infected BALB/c and IL-6 –/– mice. Data are representative of two pooled experiments each with ≥4 mice per group. B. Expression of IL-1β, IL-21 and IL-23R by quantitative PCR in whole MLNC of naïve and H. polygyrus-infected BALB/c and IL- 6 –/– mice day 7 post-infection. Data shown is one experiment representative of two independent repeats with n≥3 mice/group, where the quality of cDNA was found to be low by analysis of the Tm dissociation curve, these samples were excluded from analysis. C. Helios expression within the MLNC CD4 + Foxp3 + population in relation to correlate CD25, CD44, ICOS and Ki67 expression at steady state or at day 5 following H. polygyrus infection. D. Correlations between Helios expression and CD45RB, GATA-3, OX-40 and Foxp3 expression within the same populations Data in C and D are pooled from one experiment with naïve and infected BALB/c and IL-6 - /- and are representation of 2 repeats with n≥4 mice/group. E. Intracellular cytokine staining of MLNC CD4 + Foxp3 + T cell populations at steady state or day 7 following H. polygyrus infection for IL2, IL-10 and IL-17 in mice deficient in IL-6.. Data depicted is one experiment representative of two repeats performed with n≥3 mice/group. F. Intracellular cytokine staining of CD4 + MLNC and antigen-specific restimulation of whole MLNC to determine proportions and levels of IL-13 in mice deficient in IL-6 following administration of an anti-IL-2:IL-2 complex, representing the same experiments as in E. G. Eosinophilia in the MLNC of IL-6 –/– mice receiving anti-IL- 2:IL-2 complex administration or control treatments in the same experiments. For panels A, E, F and G, data were analysed by 1-way ANOVA, p<0.05 (*), <0.01 (**), <0.001 (***) or not significant (ns) indicated. For panels C and D, correlation analysis was performed by linear regression analysis with a x and y-intercept of 0 and using a Pearson test with a 95% interval to generate an r 2 value as shown. **Figure S2**. Gating strategy for experiments presented in Fig. 2 Lettering corresponds to the panels in Fig. 2, showing for (A) intracellular IL-4, IL- 10 and IL-13; (F) SiglecF; and (G) non-B-non-T ILC2 cells. **Figure S3**. Gating strategy for experiments presented in Fig. 4 Lettering corresponds to the panels in Fig. 4, showing for (A-K) Foxp3 and Helios; and (M) Foxp3 and KJ126. **Figure S4**. Gating strategy for experiments presented in Fig. 5 Plots corresponds to the panels in Fig. 5 showing Foxp3 and Helios.

Peer review correspondence
