# Supplementary material for: IL-6 controls susceptibility to helminth infection by impeding Th2 responsiveness and altering the Treg phenotype in vivo
Source: Eur J Immunol. 2013 Oct 8;44(1):150–61. doi: 10.1002/eji.201343746 (PMC3992848; doi:10.1002/eji.201343746)

European Journal of Immunology

Supporting Information

for

DOI 10.1002/eji.201343746

Katherine A. Smith and Rick M. Maizels

IL-6 controls susceptibility to helminth infection by impeding Th2 responsiveness and altering the Treg phenotype in vivo

© 2013 The Authors. European Journal of Immunology published by Wiley-VCH Verlag GmbH & Co. KGaA, Weinheim.

Supplementary Information

Supplementary Figure 1

A. Th17 responses during active *N. brasiliensis* infection in IL-6-deficient BALB/c mice. BALB/c and IL-6$^{-/-}$ mice were infected with 250 L3 of *N. brasiliensis* s.c. and 7 days later, MLNC recovered for intracellular cytokine staining and flow cytometry. Representative FACS staining of IL-17 versus IFN-γ (left panel) and percentage of IL-17^{+} cells (right panel) within the CD4^{+} population of naïve and *N. brasiliensis* infected BALB/c and IL-6$^{-/-}$ mice. Data are representative of two pooled experiments each with ≥4 mice per group. **B.** Expression of IL-1β, IL-21 and IL-23R by quantitative PCR in whole MLNC of naïve and *H. polygyrus*-infected BALB/c and IL-6$^{-/-}$ mice day 7 post-infection. Data shown is one experiment representative of two independent repeats with n≥3 mice/group, where the quality of cDNA was found to be low by analysis of the Tm dissociation curve, these samples were excluded from analysis. **C.** Helios expression within the MLNC CD4^{+}Foxp3^{+} population in relation to correlate CD25, CD44, ICOS and Ki67 expression at steady state or at day 5 following *H. polygyrus* infection. **D.** Correlations between Helios expression and CD45RB, GATA-3, OX-40 and Foxp3 expression within the same populations Data in C and D are pooled from one experiment with naïve and infected BALB/c and IL-6$^{-/-}$ and are representation of 2 repeats with n≥4 mice/group. **E.** Intracellular cytokine staining of MLNC CD4^{+}Foxp3^{+} T cell populations at steady state or day 7 following *H. polygyrus* infection for IL2, IL-10 and IL-17 in mice deficient in IL-6.. Data depicted is one experiment representative of two repeats performed with n≥3 mice/group. **F.** Intracellular cytokine staining of CD4^{+} MLNC and antigen-specific restimulation of whole MLNC to determine proportions and levels of IL-13 in mice deficient in IL-6 following administration of an anti-IL-2:IL-2 complex, representing the same experiments as in E. **G.** Eosinophilia in the MLNC of IL-6$^{-/-}$ mice receiving anti-IL-2:IL-2 complex administration or control treatments in the same experiments. For panels A, E, F and G, data were analysed by 1-way ANOVA, $p<0.05$ (*), <0.01 (**), <0.001 (***) or not significant (ns) indicated. For panels C and D, correlation analysis was performed by linear regression analysis with a x and y-intercept of 0 and using a Pearson test with a 95% interval to generate an r^2 value as shown.

Supplementary Figure 2

Gating strategy for experiments presented in Figure 2

Lettering corresponds to the panels in Figure 2, showing for (A) intracellular IL-4, IL-10 and IL-13; (F) SiglecF; and (G) non-B-non-T ILC2 cells.

Supplementary Figure 3

Gating strategy for experiments presented in Figure 4

Lettering corresponds to the panels in Figure 4, showing for (A-K) Foxp3 and Helios; and (M) Foxp3 and KJ126.

Supplementary Figure 4

Gating strategy for experiments presented in Figure 5

Plots corresponds to the panels in Figure 5 showing Foxp3 and Helios.

Smith et al., Suppl. Figure 1

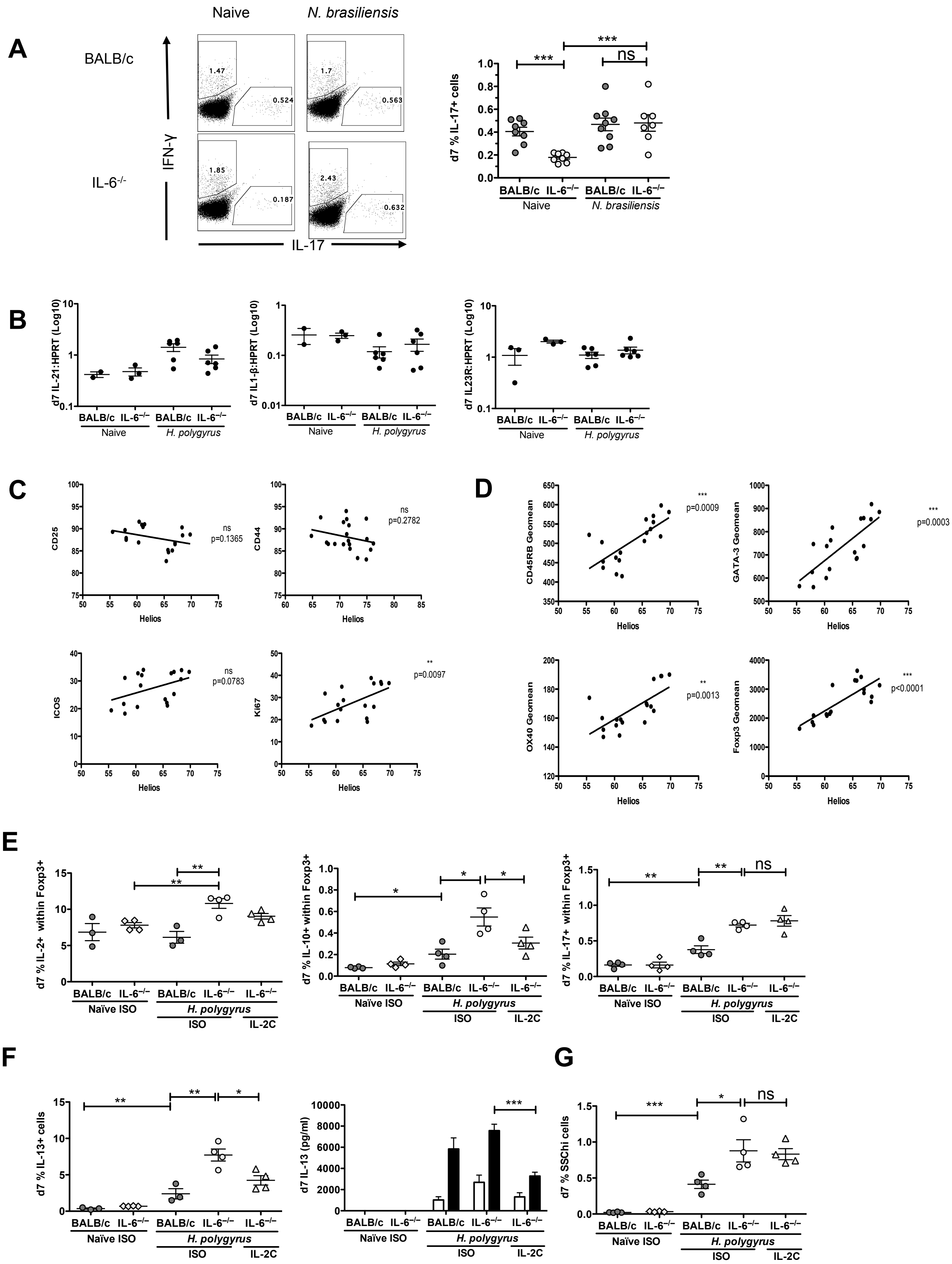

Smith et al., Suppl. Figure 2

Panel A

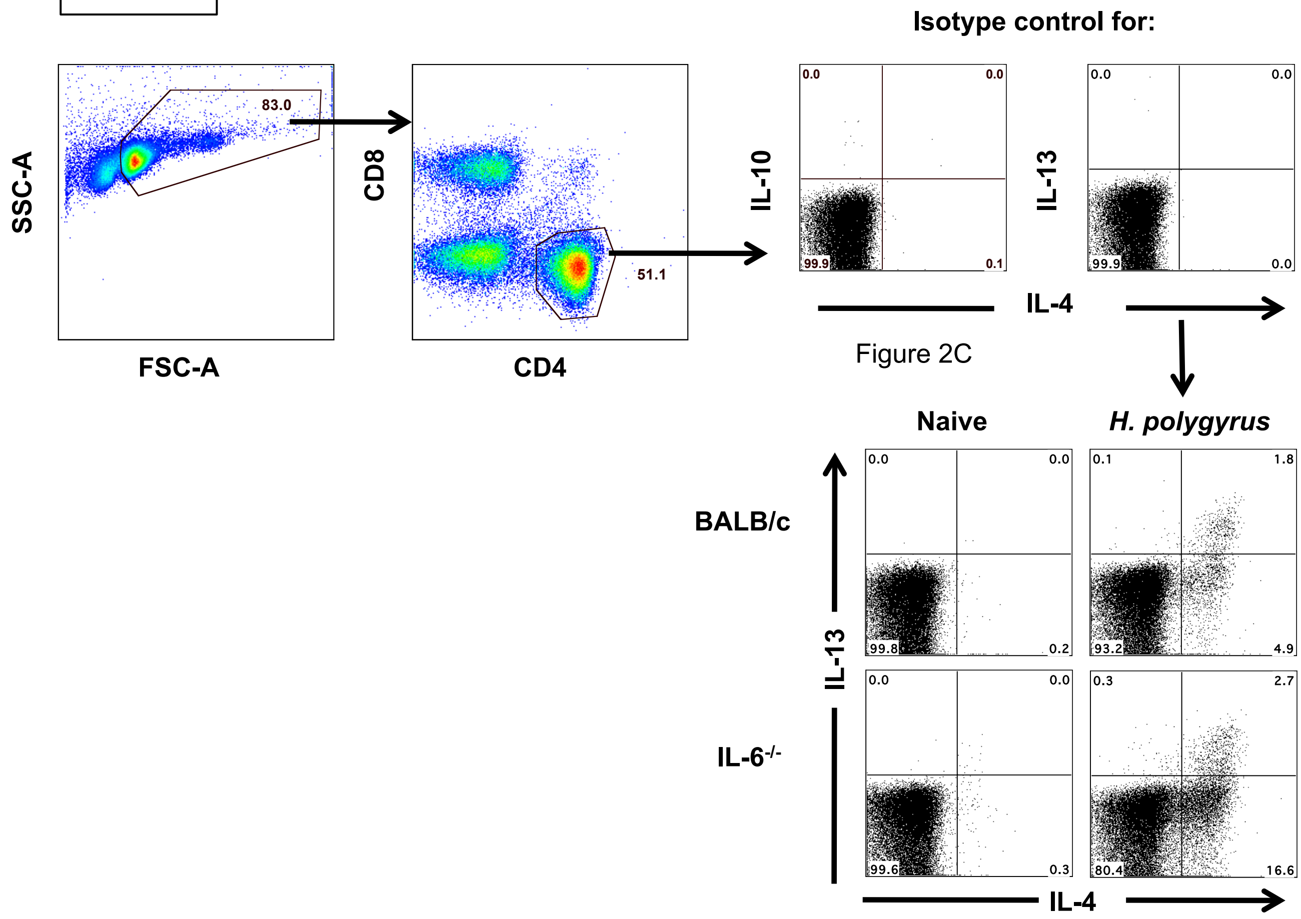

Panel F

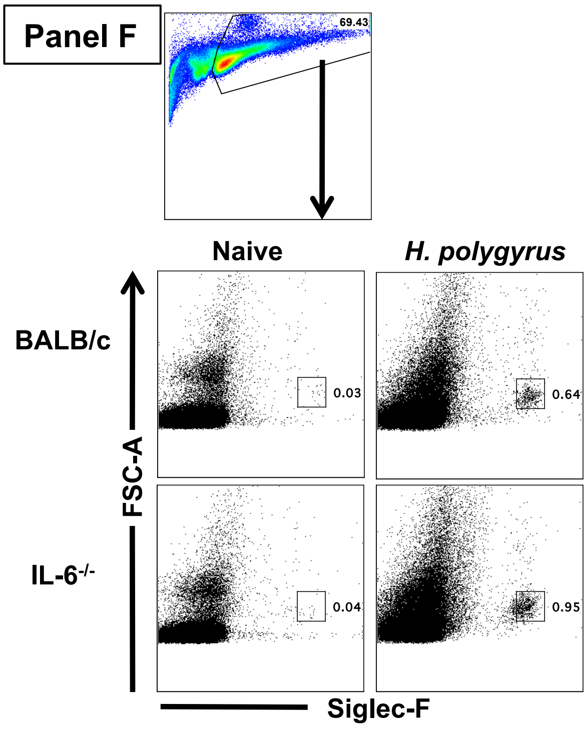

Smith et al., Suppl. Figure 2 (cont'd)

Panel G

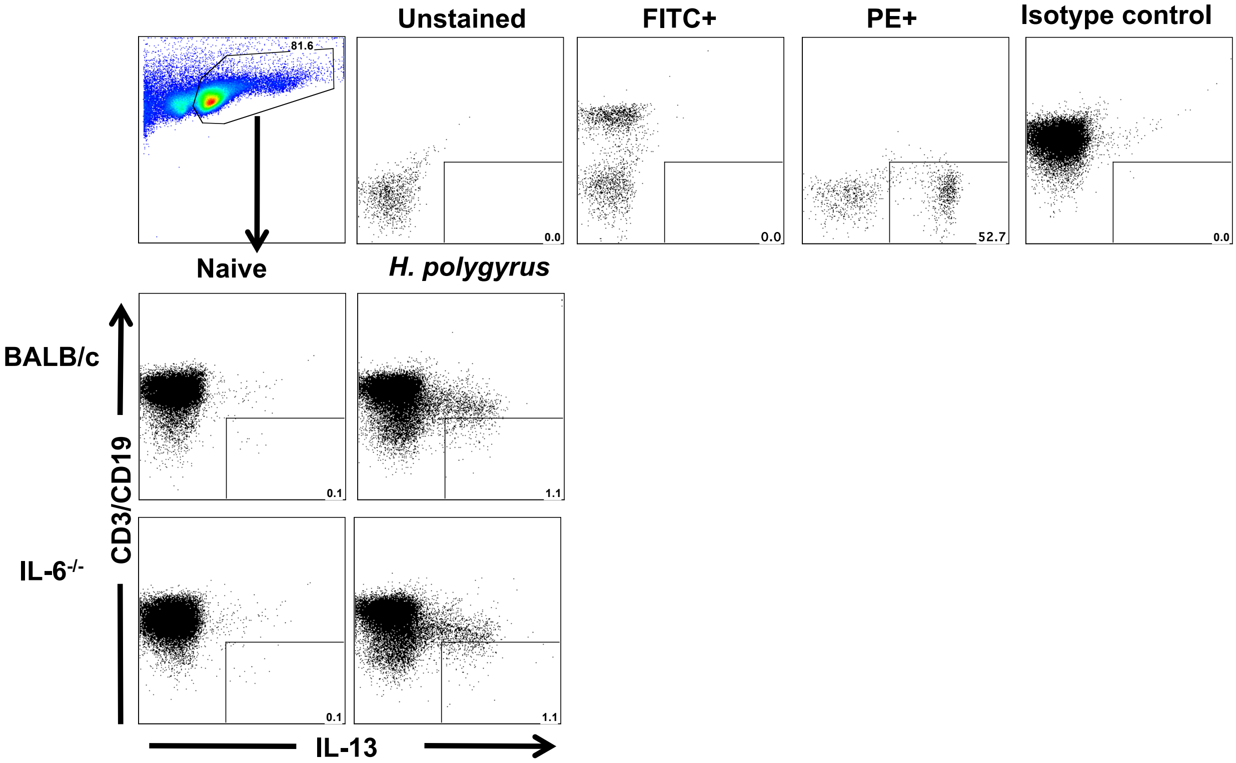

Smith et al., Suppl. Figure 3

Panels A-K

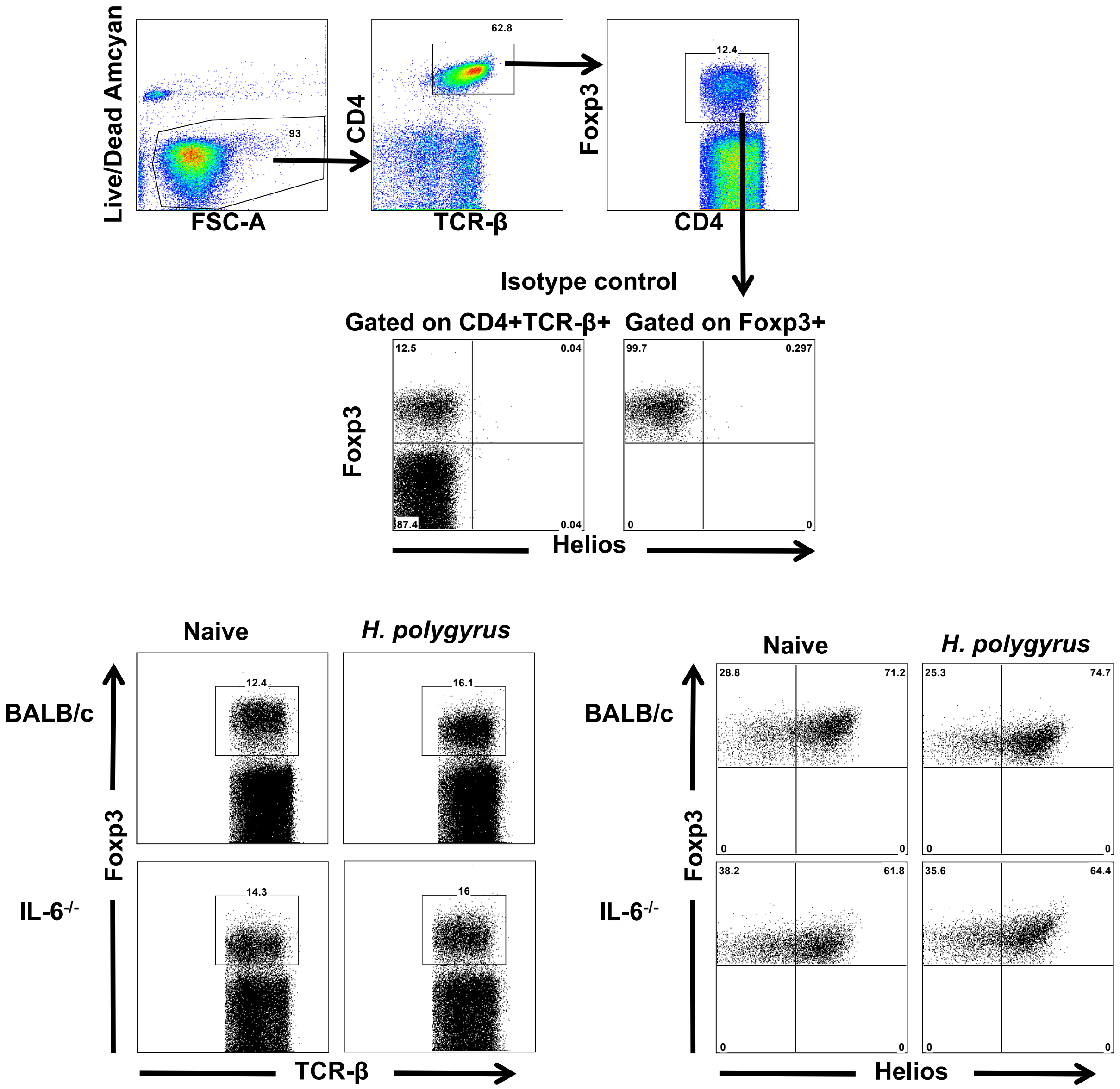

Smith et al., Suppl. Figure 3 (cont'd)

Panel M

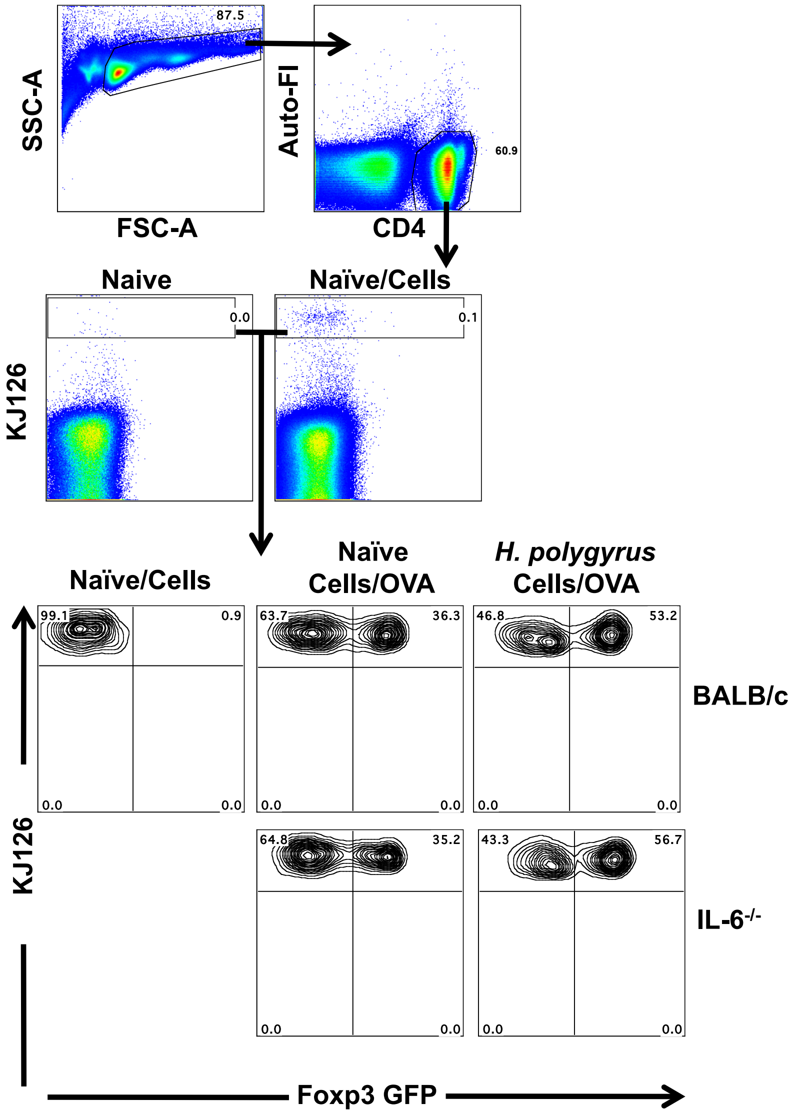

Smith et al., Suppl. Figure 4

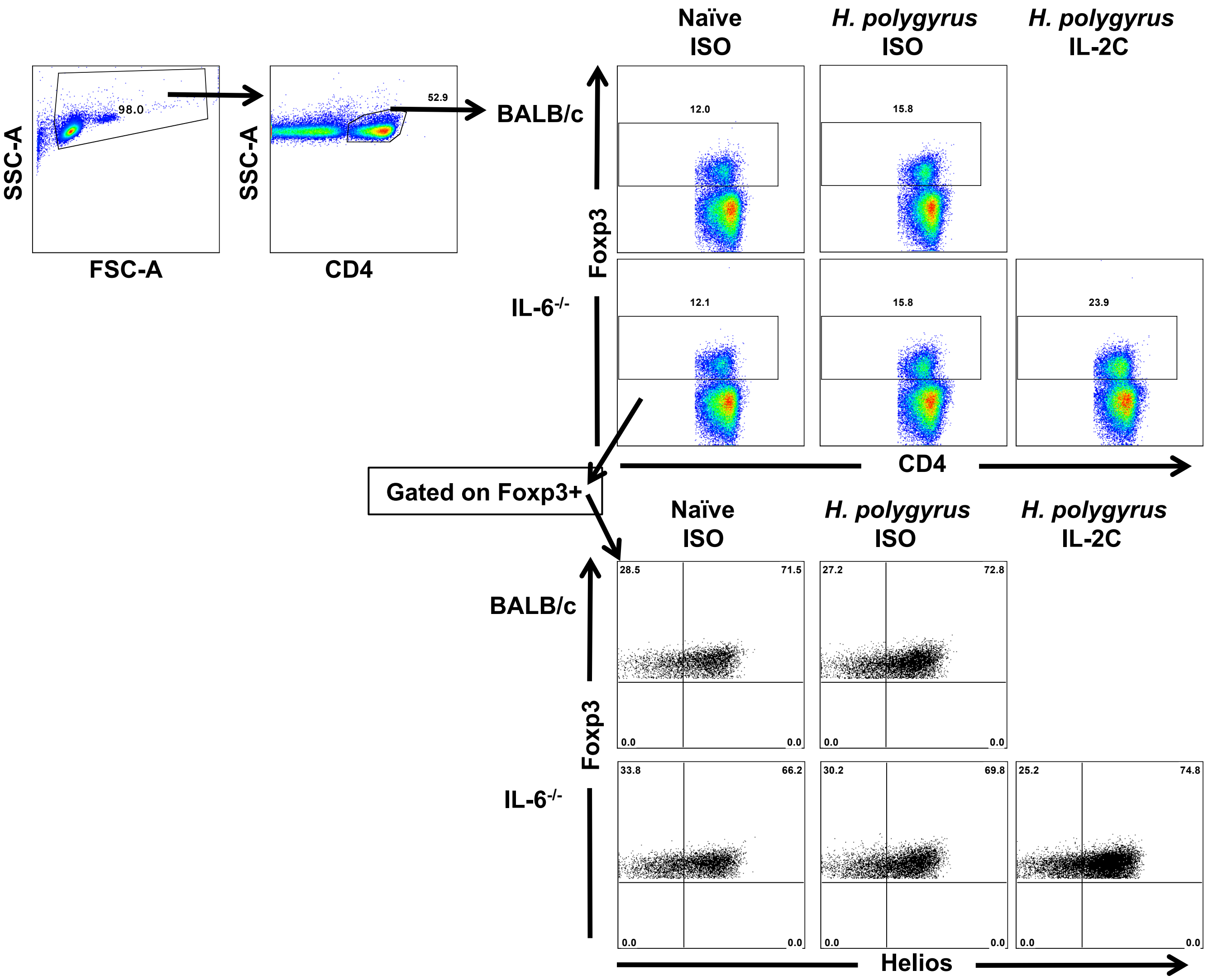

Supplement: Supplementary file 1 [file eji0044-0150-sd1.pdf]
