# Supplementary material for: IL-6 controls susceptibility to helminth infection by impeding Th2 responsiveness and altering the Treg phenotype in vivo
Source: Eur J Immunol. 2013 Oct 8;44(1):150–61. doi: 10.1002/eji.201343746 (PMC3992848; doi:10.1002/eji.201343746)
Supplement: Supplementary file 2 — Peer review correspondence [file eji0044-0150-sd2.pdf]

European Journal of Immunology

Peer review correspondence

IL-6 controls susceptibility to helminth infection by impeding Th2 responsiveness and altering the regulatory T-cell phenotype in vivo

Katherine A. Smith and Rick M .Maizels

Corresponding author: Rick M. Maizels, Institute of Immunology and Infection Research, University of Edinburgh

---

Review Timeline:	Submission date:	27 May 2013
	Editorial decision:	28 June 2013
	Revision received:	1 August 2013
	Accepted:	1 October 2013

Handling Executive Committee member: Dr. Andrea Cooper

Please note that the correspondence below does not include the standard editorial instructions regarding preparation and submission of revised manuscripts, only the scientific revisions requested and addressed.

First Editorial Decision – 28 June 2013

Dear Dr. Maizels,

Manuscript ID eji.201343746 entitled "IL-6 controls susceptibility to helminth infection by impeding Th2 responsiveness and altering Treg phenotype in vivo", which you submitted to the European Journal of Immunology, has been reviewed. The comments of the referees are included at the bottom of this letter.

Although the referees have recommended publication, some revisions to your manuscript have been requested. Therefore, I invite you to respond to the comments of the referees and revise your manuscript accordingly.

If the revision of the paper is expected to take more than three months, please inform the editorial office. Revisions taking longer than six months may be assessed by new referees to ensure the relevance and timeliness of the data.

Once again, thank you for submitting your manuscript to European Journal of Immunology. We look forward to receiving your revision.

European Journal of Immunology

Yours sincerely,
Karen Chu

on behalf of Dr. Andrea Cooper

Dr. Karen Chu
Editorial Office
European Journal of Immunology
e-mail: ejied@wiley.com
www.eji-journal.eu

****

Reviewer: 1

Comments to the Author
The manuscript "IL-6 controls susceptibility to helminth infection by impeding Th2 responsiveness and altering Treg phenotype in vivo" by Smith and Maizels focuses on the role of IL-6 in shaping the balance of regulatory T cells and effector T cells during helminth i.e. Heligmosomoides polygyrus infection.
IL-2 and TGF-beta promote Treg-cell development whereas IL-6 and TGF-beta promote Th17-cell development. Comparing immune response and host defence in wild type and IL-6 knockout mice, the authors record an unexpected result. IL-6 deficiency did not increase the number of Treg and subsequently susceptibility to infection, as one would expect if the Th17 diversion is blocked in the absence of IL-6 thus favouring Treg development in the presence of remaining TGF-beta. In contrast, the authors observe increased resistance and improved Th2 immune response as shown by intracellular cytokine staining and in vitro re-stimulation of lymphocytes in the absence of IL-6.
Constitutively cytokine deficient mice often display in vivo compensation effects as other immunological effecters are up or down-regulated during ontogeny to compensate the lack of a given cytokine/effector molecule. Moreover, development of the immune system in the absence of IL-6 affects multiple relevant effectors. The authors recognize this and perform a very important control experiment that is to reproduce the unexpected phenotype of IL-6 knockout mice in wild type mice that received neutralizing mAb to IL-6 during infection.
As expected, the Th17 frequency was lower in naïve IL-6 deficient mice but reached wild type levels during infection with H. polygyrus and another gastrointestinal nematode, Nippostrongylus brasiliensis. The IL-6 independent Th17 expansion could not be tracked down to changes in other Th17 promoting factors such as IL-21, IL-23 and IL-1. IL-17 was not important for host defence during H. polygyrus infection as was shown by in vivo IL-17 neutralization.

European Journal of Immunology

Further seeking for the underlying mechanism of increased resistance in IL-6 deficient mice, the authors analysed the phenotype of Treg expanding in the presence and absence of IL-6 and reported differences. Although no black and white results were obtained, the accumulated differences in GATA-3, Foxp3, Helios and cytokine expression within the regulatory T cell population suggested that Treg expanding in vivo in the absence of IL-6 may be less stable and more prone to re-diversion towards a T effector phenotype.
In this context the authors performed the second important control. The showed that forced expansion of the Treg population that was achieved by application of anti-IL-2:IL-2 complexes reduced Th2 responses and increased parasite burden in IL-6 knockout mice. Thereby the authors established that indeed increased numbers of effective "stable" Treg cells counteracted initiation of Th2 response in their system also in the absence of IL-6.

This study does not provide direct causative links between the changed phenotype of the Treg cells expanding in nematode infected mice in the absence of IL-6 and the improved Th2 response as well as reduced worm burden. It does, however, provide strong correlative evidence that is presented and discussed adequately. The two central controls (see above) and other minor controls experiments ruled out a central contribution of other mechanisms than changed Treg-cell phenotype for the effects of IL-6 deficiency in H. polygyrus infected mice (with the exception of IgE, see below). The accumulated correlative evidence, therefore, is rather convincing and of high interest for the scientific community showing contribution of IL-6 on Treg-cell stability (presumably nTreg-cell stability) and phenotype during gastrointestinal nematode infection.

The following critique could be addressed:

1) The authors show that antigen-specific IgE is increased in IL-6 deficient mice day 21 of infection (and state that other isotypes were unchanged). Could this increased IgE response contribute to resistance? Could the authors comment on the role of IgE in immune response to H. polygyrus in general and the putative role of IgE in improved resistance in IL-6 ko mice.

2) The authors show that IL-17 is not needed for immunity to H. polygyrus by in vivo neutralization (applying 50 microgram mAb per application which is almost five fold lower than the usual amount used for neutralization). Could the authors please cite the study that demonstrated biologically relevant IL-17 depletion with the depletion regime they used and mention the possibility that depletion may have failed or otherwise provide a depletion control?

3) The authors are a little bit inconsistent with the provision of example dotplots for flow cytometry. I would appreciate a supplementary figure displaying the gating strategy and example dotplots for all measurements used to generate results displayed as graphs even in addition to the dotplots the authors introduced in the figures. Here especially in figure 5A the right panel where very, very mild but statistically

European Journal of Immunology

significant differences in frequency of Foxp3+Helios+ Treg are shown and for identification of ILC-2 in Figure 2G the complete gating strategy would be informative to the reader.

For Figure 1, Figure 2 and Figure 5: could the authors please state if one representative experiment or the pooled data of all repeats are shown for each panel
Fig 4.

Page 11 line 30 ff one citation is unformatted.

Reviewer: 2

Comments to the Author
The conclusions of the authors would be enhanced if they could show to what levels that IL-6 is actually induced following an Hp infection and the source of the immunoregulatory IL-6 in the Hp infection. Also whether experiments were included that involved administration of IL-6 to the IL-6-/- mice to restore a susceptible phenotype. The pleiotropic nature of IL-6 bedevils interpretation of whether the IL-6 is acting at the level of the DC, Macrophage or Treg cells as suggested, some discussion of these issues could help clarify the position and conclusions the authors have taken.

Reviewer: 3

Comments to the Author
The manuscript from Drs. Smith and Maizels is a nice study on the role of IL-6 during acute helminth infections and its impact on host immune responses. I have only minor criticisms and comments which would make the manuscript easier to read. My only concern and perhaps irritation is that the authors constantly change the day in which the analysis was performed without addressing why they present the data from that day and only show data from 1 representative experiment. The authors should try to re-word the manuscript to inform the reader why they chose those days and perhaps pool some of the data together in the graphs.

Figure 1.
For ease of reading the authors should add the day of analysis on the y-axis of the graphs.
Do the authors have data on the granuloma size on day 28? Or can they comment on this matter, ie whether residual granulomas are still present, whether they are still enlarged or reduced to WT levels.
Where the adult worms equally male and female, ie, can the authors rule out that the reduction in eggs was not due to lack of fecund females.

European Journal of Immunology

I assume that the drop in adult worm burden between days 14 and 28 is normal? Can the authors comment on whether it was a significant drop? Do the authors know at what time-point the worms in the IL-6 ko mice were eliminated?

Figure 2.

In A), since the authors make a point about significance between naive and infected WT mice I think they should also provide stats between naive and infected ko groups.

Can the authors comment whether the increased cytokine levels in infected IL-6 ko mice were also elevated on days 14 and 28 when they did the parasite analysis, especially when they link their hypothesis and findings to the granuloma data.

Did the authors perform IL-5 staining?

Although I am fully in agreement that IL-10 can also be classified as a Th2 cytokine many do not like this term. Thus, it would be good if the authors modified their text to "Th2 cytokines IL-4 and IL-13 and the regulatory cytokine IL-10 by intracellular....".

It is not necessary to have the cytokines in brackets on the y axis of your graphs it looks confusing whether both cytokine and concentration are in their own brackets.

Try and make it clearer that 2E depicts levels of specific antibody

The quality of 2C is low, I would suggest to increase its quality and substantiate your findings by adding in a graph of IL-4+IL-10+ cells. It would be good to bring this point also into the discussion especially since research on helminth-infected individuals has also shown this same phenomena (Mitre et al., 2008).

I think it would strengthen your argument if you comment on the parasite levels of your 20F3 mice, especially whether worm burden had vanished by day 28.

Can the authors comment on why IL-6 ko mice had higher levels of IgE on day 21 despite having lower worm burden levels?

Figure 3

The text to Fig. 3A is misleading. You actually measured IL-17 levels on days 5, 7, 14 and 28 not just day 5 (line 42). You state that the fold increase in IL-6 ko is much higher than WT which is nicely depicted in B. However in A you show data from day 5 and this is not as convincing (even thought I see it is confirmed in B). However, if you really want to visually convince the readers then I would recommend you should show your FACS plots and graphs in A from day 7 - or better day 5 and 7.

In addition, was the data significant between naive and WT infected mice, since you make a point about significance between naive and infected ko groups.

I would additionally add in IL-4+ T cell data to C to strengthen your statements, since you only mention in the text that antibody treatment did not alter Th2 responses (page 6 lines 19-23ish) but do not show data nor state (data not shown).

Did the authors not have access to IL-17 deficient mice?

European Journal of Immunology

Figure 4

The Treg data is really nice and its really interesting that the IL-6 ko mice have higher duel-positive cells. However, again here A-C are day 5 and D-E day 7. So, can the authors comment whether the dual positive cells are observed only on day 5 and does the elevated Treg number seen on day 5 hold also on day 7. Did the authors analyze Treg or duel positive cells at later timepoints on day 14 or 28. IL-17 has been associated with granuloma size so perhaps the elevated granuloma size is due to higher proportions of these cells. As an idea for the future, one could think of doing IHC staining on duel positive cells.

A question that is often asked in such studies on Treg/IL-17+ cells is whether these cells also up-regulated RORgt. Did the authors also check the transcription factor levels?

Minor points

The symbols for gamma and beta (IFN, TGF and IL-1) are missing throughout the script and also on the y-axis of Fig.3A.

"-/-" should be superscripted in the script and also in the figures.

H. polygyrus should be in italics throughout the manuscript and in the figures. Also sometimes in the text there is a space between H and poly and sometimes not.

In the flow cytometry image of Fig. 3A, the vertical quadrant lines are missing in the right side panel.

The "=" sign, denoting the number of mice is missing throughout the figure legends.

Please note throughout the results text section where the cells for FACS and re-stimulation came from.

page 8 line 10, missing a hyphen in IL-6 and in IL-2C line 10 page 9. This also occurs in Fig. 5D graph legend.

The authors should improve the figure legends in the supplementary section. For example, no statistical applications are mentioned, the days of anaylsis are missing in and where cells originated from in C-through G. Please state what SSChi cells mean in Figure SG.

Remove" -" page 10 line 8.

First Revision – authors' response – 1 August 2013

Point-by-point response

Thankyou for your email dated 28 June 2013 with the positive decision on our manuscript, subject to suitable revisions. We very much appreciated the thoughtful comments provided by the Reviewers. In response, we have fully revised the original submission and clarified the manuscript in a number of respects, as set out in detail below.

Reviewer #1:

1) The authors show that antigen-specific IgE is increased in IL-6 deficient mice day 21 of infection (and state that other isotypes were unchanged). Could this increased IgE response contribute to resistance? Could the authors comment on the role of IgE in immune response to H. polygyrus in general and the putative role of IgE in improved resistance in IL-6 ko mice.

We thank the reviewer for their comments. IgE is indeed induced in response to primary and secondary *H. polygyrus* infection and it is possible that it may contribute to heightened resistance in IL-6$^{-/-}$ mice by binding the high affinity IgE receptor on mast cells and basophils and inducing their activation and release of cytokines and chemical effectors. However, experiments in IgE deficient mice have shown that IgE is not required for a protective response following secondary infection and that only passive transfer of IgG can reduce adult worm burden following primary infection with *H. polygyrus* (McCoy KD *et al.*, 2008 *Cell Host & Microbe* **4**:382). We therefore feel that the elevated IgE simply reflects the stronger Th2 milieu in infected IL-6$^{-/-}$ mice, rather than thinking that higher levels of IgE directly contribute to the improved resistance following primary infection of these mice. *We have now added a paragraph to the discussion on page 10, line 27 of the manuscript discussion to clarify the role of IgE in the immune response to H.polygyrus and its putative role in improved resistance in IL6KO mice.*

2) The authors show that IL-17 is not needed for immunity to H. polygyrus by in vivo neutralization (applying 50 microgram mAb per application which is almost five fold lower than the usual amount used for neutralization). Could the authors please cite the study that demonstrated biologically relevant IL-17 depletion with the depletion regime they used and mention the possibility that depletion may have failed or otherwise provide a depletion control?

We thank the reviewer for this comment. 50ug of this clone has been effectively used to neutralize IL-17 in a mouse model of allergic asthma (Hellings PW et al., 2003 Am J Respir Cell Mol Biol 28:42-50, Besnard AG et al., 2011 Am J Respir Crit Care Med 183:1153-63 and Draper DW et al., 2012 JI 188:5327-5336). Similar to

European Journal of Immunology

the protocols used in these papers, we delivered 50μg intraperitoneally four times every three days, equivalent to a total administration of 200μg. *In order to clarify this further, we have cited these studies and stated the total amount of antibody used in our study in the material and methods section on page 13, line 24.*

3) The authors are a little bit inconsistent with the provision of example dotplots for flow cytometry. I would appreciate a supplementary figure displaying the gating strategy and example dotplots for all measurements used to generate results displayed as graphs even in addition to the dotplots the authors introduced in the figures. Here especially in figure 5A the right panel where very, very mild but statistically significant differences in frequency of Foxp3+Helios+ Treg are shown and for identification of ILC-2 in Figure 2G the complete gating strategy would be informative to the reader.

We appreciate the Reviewer's point and accordingly provide new Supplementary Figures 2, 3 and 4 presenting the gating strategy used for the main paper Figures 2, 4 and 5 respectively.

For Figure 1, Figure 2 and Figure 5: could the authors please state if one representative experiment or the pooled data of all repeats are shown for each panel Fig 4.

We have full detailed this point in all 5 Figure Legends.

Page 11 line 30 ff one citation is unformatted.

We have corrected this mistake.

Reviewer #2:

The conclusions of the authors would be enhanced if they could show to what levels that IL-6 is actually induced following an Hp infection and the source of the immunoregulatory IL-6 in the Hp infection. Also whether experiments were included that involved administration of IL-6 to the IL-6-/- mice to restore a susceptible phenotype. The pleiotropic nature of IL-6 bedevils interpretation of whether the IL-6 is acting at the level of the DC, Macrophage or Treg cells as suggested, some discussion of these issues could help clarify the position and conclusions the authors have taken.

We thank the Reviewer for their comments, indeed the precise cellular source of IL-6 that contributes to this phenotype, is an area that particularly interests us for further research. Our preliminary evidence suggests that the production of IL-6, and the cell source of IL-6, changes dramatically over the course of infection, however, we felt that the word limitations for this publication limited discussion of this in full. For this reason, we take the approach of focusing on the interaction of IL-6 with T cell populations, as changes to effector and regulatory responses are the most evident in this system. Given the highly dynamic nature of differing amounts of IL-6 production over time, we considered this highly challenging to replicate by injection of rIL-6 *in vivo*. *To address the concerns of the Reviewer, we have further highlighted our interest in defining the cellular source of IL-6 by the addition of a sentence on page 12, line 25.*

Reviewer #3:

European Journal of Immunology

The manuscript from Drs. Smith and Maizels is a nice study on the role of IL-6 during acute helminth infections and its impact on host immune responses. I have only minor criticisms and comments which would make the manuscript easier to read. My only concern and perhaps irritation is that the authors constantly change the day in which the analysis was performed without addressing why they present the data from that day and only show data from 1 representative experiment. The authors should try to re-word the manuscript to inform the reader why they chose those days and perhaps pool some of the data together in the graphs.

We thank the Reviewer for their comments and have added the time-point of analysis to the y-axis in order to clarify this point. We have also added a sentence to the results section on page 4, line 7 to clarify why we looked at day 14 post-infection and have added an explanation for subsequent analysis of parasitology at day 28 post-infection on line 10-11. We also modified "earlier weeks" to "initial priming phases" on page 4, line 21 to inform the reader that this figure focuses on the early immune response following infection and added "longer-term" to page 5, line 7 to explain analysis of antigen-specific antibody at day 21 post-infection. We have clarified that eosinophilia is also significantly higher in IL6KO at day 7 post-infection and inserted a reference to Supplementary Figure 1G, which demonstrates this on page 5, line 14 and 17 and inserted "day 5" in between early and induction of ILCs on page 5, line 18. We have also inserted a sentence on page 7, line 9-10 to explain why we looked at day 7 for Treg cytokine responses and a sentence in line 21 as to why we then assess Treg phenotype at day 5 post-infection.

For ease of reading the authors should add the day of analysis on the y-axis of the graphs.

We have now included the day of analysis on the y-axis of all the graphs, and agree that this greatly clarifies the presentation of the data.

Do the authors have data on the granuloma size on day 28? Or can they comment on this matter, ie whether residual granulomas are still present, whether they are still enlarged or reduced to WT levels.

At day 28 post-infection, residual granulomas are present but at a lower frequency than at day 14 in both strains (Figure below). However, the pattern seen at day 14 is still reflected at day 28 i.e. granuloma frequency is significantly higher in the IL6KO than the WT. *We have now included a sentence addressing this matter in the results section on page 4, line 13.*

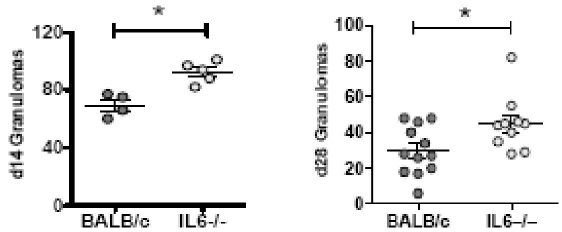

Were the adult worms equally male and female, ie, can the authors rule out that the reduction in eggs was not due to lack of fecund females. I assume that the drop in adult worm burden between days 14 and 28 is normal? Can the authors comment on whether it was a significant drop? Do the authors know at what time-point the worms in the IL-6 ko mice were eliminated?

European Journal of Immunology

As worm burdens were equivalent between strains at day 14 post-infection, we do not believe that there was a selective loss of a specific sex of worm, rather that pairing and fitness was affected, ultimately impacting on egg burden. In BALB/c mice the drop in adult worm burden is normal as the adult emerges into the lumen and is gradually expelled through the small, then large intestine. Mice cannot be individually tracked to perform a paired-comparison and assess significance in the drop between worm burden at day 14 and d28, as these can only be measured post-mortem. Worms were assessed in one experiment at day 21 post-infection and found to be equivalent in both strains (Figure 2 below), suggesting that eventual elimination from the intestine occurs between day 21 and day 28 post-infection.

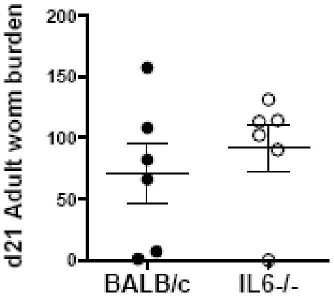

Figure 2.
In A), since the authors make a point about significance between naive and infected WT mice I think they should also provide stats between naive and infected ko groups.

We have performed statistical testing and added the appropriate significance to this figure.

Can the authors comment whether the increased cytokine levels in infected IL-6 ko mice were also elevated on days 14 and 28 when they did the parasite analysis, especially when they link their hypothesis and findings to the granuloma data.

Th2 cytokine responses were increased in infected IL-6KO mice at day 14 and day 28 post-infection both by intracellular staining and antigen-specific re-stimulation. This reflects the worm burden in these mice and correlates with the granuloma data. *We have included a comment reflecting this on page 4, line 9 and 15*.

Did the authors perform IL-5 staining?

We confirm that intracellular staining of CD4+ T cells with IL-5 was performed but that it was not significantly different between strains following infection at day 5 post-infection, however by day 14 post-infection it reflected the eosinophil response shown in 2F.

Although I am fully in agreement that IL-10 can also be classified as a Th2 cytokine many do not like this term. Thus, it would be good if the authors modified their text to "Th2 cytokines IL-4 and IL-13 and the regulatory cytokine IL-10 by intracellular....".

We have modified this sentence on page 4, line 27

It is not necessary to have the cytokines in brackets on the y axis of your graphs it

European Journal of Immunology

looks confusing whether both cytokine and concentration are in their own brackets.

We have removed these brackets from the y axis of the cytokine data.

Try and make it clearer that 2E depicts levels of specific antibody.

We have clarified this point in the Figure Legend for Figure 2 by the addition of "antigen-specific" before "IgE" and modifying "HES-specific" to "antigen-specific" on page 5, line 7 of the results section.

The quality of 2C is low, I would suggest to increase its quality and substantiate your findings by adding in a graph of IL-4+IL-10+ cells. It would be good to bring this point also into the discussion especially since research on helminth-infected individuals has also shown this same phenomena (Mitre et al., 2008).

In response to the Reviewer's comments, we have replaced Figure 2C with a higher quality graph of CD4+ T cells expressing IL-4+IL-10+ cells. We have also added a sentence referring to the role of IL-10+CD4+ T cells in this infection setting, and related this to helminth-infected individuals in the discussion on page 10, line 22

I think it would strengthen your argument if you comment on the parasite levels of your 20F3 mice, especially whether worm burden had vanished by day 28.

Whilst we do agree with the Reviewer that this experiment would strengthen our argument, it is not feasible to perform longer-term neutralization studies due to the anticipated neutralization of this rat anti-mouse monoclonal antibody by the mouse immune system and the prohibitive economic costs of delivering 200ug of antibody per mouse every 2 days, over a course of 28 days.

Can the authors comment on why IL-6 ko mice had higher levels of IgE on day 21 despite having lower worm burden levels?

The IgE response to helminth infection is T-cell dependent and dependent on IL-4 (Ubran JF et al., 1991 PNAS 88:5513), therefore heightened IgE reflects the heightened Th2 response in these mice. *We have addressed this on Page 10 in the paragraph on the role of IgE in the immune response to H.polygyrus that we have added in order address the comments of reviewer 1.*

Figure 3
The text to Fig. 3A is misleading. You actually measured IL-17 levels on days 5, 7, 14 and 28 not just day 5 (line 42). You state that the fold increase in IL-6 ko is much higher than WT which is nicely depicted in B. However in A you show data from day 5 and this is not as convincing (even though I see it is confirmed in B). However, if you really want to visually convince the readers then I would recommend you should show your FACS plots and graphs in A from day 7 - or better day 5 and 7.
In addition, was the data significant between naive and WT infected mice, since you make a point about significance between naive and infected ko groups.
I would additionally add in IL-4+ T cell data to C to strengthen your statements, since you only mention in the text that antibody treatment did not alter Th2 responses (page 6 lines 19-23ish) but do not show data nor state (data not shown). Did the authors not have access to IL-17 deficient mice?

We apologise for this mistake and have clarified the days of analysis of IL-17 in the results section on page 5, line 29. We have improved the resolution of the graphical image in A, so as to provide more convincing visual data for the reader. The IL-17

European Journal of Immunology

response in wild-type BALB/c increased following infection but did not reach statistical significance.
Because Figure 3 A presents both IL-17 and IFN-γ responses, we accompanied this with flow cytometry plot from day 5 cells in which we co-stained for both cytokines. In the day 7 experiments, for other reasons we co-stained IL-17 and IL-4; these data show equivalent IL-17 responses to day 5 and we provide these for the Reviewer below:

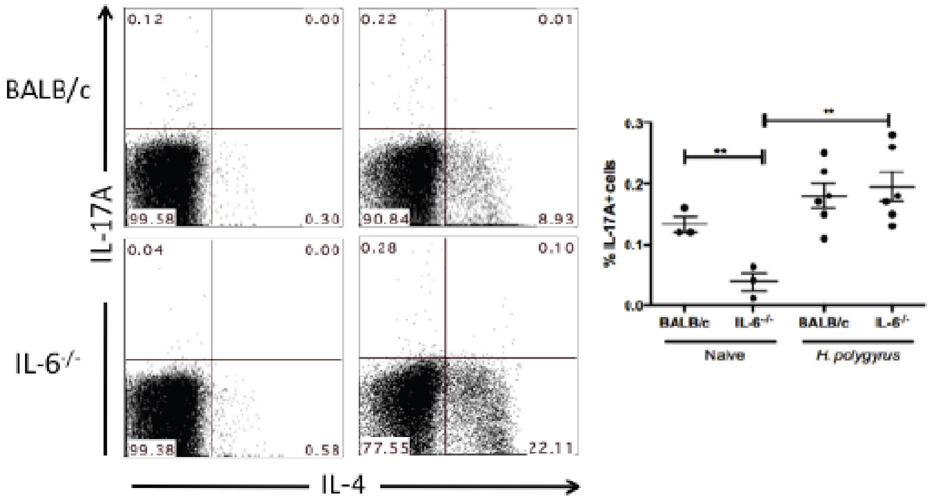

We have also modified the Figures to include a statistical analysis between naïve and infected IL-6-/- groups and added the statement "data not shown" to the results section for Figure 3C. Unfortunately we did not have access to IL-17 deficient mice, and the presence of multiple IL-17 isoforms (IL-17A, IL-17F etc) raises further issues for a genetic knockout strategy..

Figure 4
The Treg data is really nice and its really interesting that the IL-6 ko mice have higher dual-positive cells. However, again here A-C are day 5 and D-E day 7. So, can the authors comment whether the dual positive cells are observed only on day 5 and does the elevated Treg number seen on day 5 hold also on day 7. Did the authors analyze Treg or dual positive cells at later timepoints on day 14 or 28.

We thank the Reviewer for their comments. At day 5, Tregs from IL-6-/- have higher IL-17 and IL-10+ following infection, but this phenotype is more marked at day 7, when Tregs from IL-6-/- start making IL-2, along with higher levels of other cytokines as shown. As the Treg cytokine phenotype exacerbates over the course of infection, we felt it more accurate to represent Treg cytokine production at day 7, when effector T cell cytokine production was significantly increased in the IL-6-/-. *We have clarified the reasoning for this on page 7, line 13 and 25 in the manuscript*. We did not analyse cytokine production by Tregs at later points in infection, as we were interested in changes to Treg phenotype at these earlier time points, which coincided with the increased effector responses in the IL-6-/- seen by day 7 post-infection (Figure 2).

IL-17 has been associated with granuloma size so perhaps the elevated granuloma size is due to higher proportions of these cells. As an idea for the future, one could think of doing IHC staining on duel positive cells. A question that is often asked in

European Journal of Immunology

such studies on Treg/IL-17+ cells is whether these cells also up-regulated RORgt. Did the authors also check the transcription factor levels?

We thank the Reviewer for these comments and suggestions. We did also check the expression of RORγT by FACS intracellular staining. We were unable to validate correlation of expression with IL-17 positivity, however, this was most likely due to low levels of RORγT expression in the MLNC and the limitation of the antibody clone used.

Minor points

The symbols for gamma and beta (IFN, TGF and IL-1) are missing throughout the script and also on the y-axis of Fig.3A.

In all but two instances these symbols were present in the original files and hope the probelm will not recur during file conversion of the revised manuscript. *We have also double-checked throughout the manuscript and have corrected one instance of IFN-gamma to IFN-γ and one instance of IFN-beta to IFN-β that were in the Supplementary information.*

"-/-" should be superscripted in the script and also in the figures.

We have amended three instances of this mistake in the main text and within all of the Figures.

H. polygyrus should be in italics throughout the manuscript and in the figures. Also sometimes in the text there is a space between H and poly and sometimes not.

We have corrected this so that H. polygyrus (with a space) is used throughout.

In the flow cytometry image of Fig. 3A, the vertical quadrant lines are missing in the right side panel.

Again this was correct in the original file and we will check to ensure this does not recur during file conversion of the revised manuscript.

The "=" sign, denoting the number of mice is missing throughout the figure legends.

As above, is correct in the original and we will check during resubmission that the problem does not recur

Please note throughout the results text section where the cells for FACS and re-stimulation came from.

We have now amended the text and Figure Legends accordingly so that it is clear where the cells for FACS and restimulation for each experiment came from.

page 8 line 10, missing a hyphen in IL-6 and in IL-2C line 10 page 9. This also occurs in Fig. 5D graph legend.

We have corrected these and other instances of missing hyphens in the manuscript and the graph legends as well as the figures themselves.

The authors should improve the figure legends in the supplementary section. For example, no statistical applications are mentioned, the days of anaylsis are missing

in and where cells originated from in C-through G. Please state what SSChi cells mean in Figure SG.

We have improved the Supplementary section by addition of an explanation of the statistical tests and abbreviations used, clarified SSChi, as well as days of analysis and number of mice used per experiment.

Remove" -" page 10 line 8.

Here we assumed that the reviewer was referring to the incorrect use of a hyphen between "steady" and "state" and we have deleted this accordingly.

Editorial comments

Figures and legends
Please check your Figure legends thoroughly. In particular, in each figure legend please indicate the statistical test used to generate any p values described therein. Please indicate not only the number of experiments performed but clarify whether the data are representative or pooled from these experiments.

In response to Reviewer 1 we have included details in every case of the number of experiments and whether pooled data are presented, and in addition we have now specified the statistical tests used in each Figure.

Ensure that the data and the corresponding legends are consistent with one another and that all information (asterisks, arrows, other symbols etc) are explained. The legend should contain a brief description of the experimental conditions and experimental readout used, permitting readers to understand the data presented without reference to the main text. Please note that legends SHOULD NOT contain an interpretation of data i.e. you should not describe the results obtained as this belongs in the Results section.

We have ensured that all information in the Figures is explained and revised the headings for each Figure to remove any interpretative phrasing.

For FACS analysis: please show full gating strategy used. If multiple experiments use the same gating, please show this only once. If space is limited, you may wish to include this information as Supporting Information.

In response to Reviewer 1 we now provide Supplementary Figures 2-4 with the full gatng strategies as requested

We would like again to thank you and the Reviewers for constructive and supportive comments which we feel have helped enhance the manuscript further, and we hope that you will now judge the Revised paper as fully acceptable for publication in the European Journal of Immunology.

Yours sincerely

RMaiz

Rick Maizels (corresponding author) and Katherine Smith

Peer review correspondence

European Journal of Immunology

Second Editorial Decision – 4 September 2013

Dear Dr. Maizels,

It is a pleasure to provisionally accept your manuscript entitled "IL-6 controls susceptibility to helminth infection by impeding Th2 responsiveness and altering Treg phenotype in vivo" for publication in the European Journal of Immunology. For final acceptance, please follow the instructions below and return the requested items as soon as possible as we cannot process your manuscript further until all items (copyright forms etc.) are dealt with.

Please note that EJI articles are now published online a few days after final acceptance (see Accepted Articles: http://onlinelibrary.wiley.com/journal/10.1002/(ISSN)1521-4141/accepted). The files used for the Accepted Articles are the final files and information supplied by you in Manuscript Central. You should therefore check that all the information (including author names) is correct as changes will NOT be permitted until the proofs stage.

We look forward to hearing from you and thank you for submitting your manuscript to the European Journal of Immunology.

Yours sincerely,
Karen Chu

on behalf of Dr. Andrea Cooper

Dr. Karen Chu
Editorial Office
European Journal of Immunology
e-mail: ejied@wiley.com

www.eji-journal.eu
